# MitoTNT: Mitochondrial Temporal Network Tracking for 4D live-cell fluorescence microscopy data

**DOI:** 10.1371/journal.pcbi.1011060

**Published:** 2023-04-21

**Authors:** Zichen Wang, Parth Natekar, Challana Tea, Sharon Tamir, Hiroyuki Hakozaki, Johannes Schöneberg

**Affiliations:** 1 Department of Pharmacology, University of California, San Diego, San Diego, California, United States of America; 2 Department of Chemistry and Biochemistry, University of California, San Diego, San Diego, California, United States of America; North Carolina State University, UNITED STATES

## Abstract

Mitochondria form a network in the cell that rapidly changes through fission, fusion, and motility. Dysregulation of this four-dimensional (4D: x,y,z,time) network is implicated in numerous diseases ranging from cancer to neurodegeneration. While lattice light-sheet microscopy has recently made it possible to image mitochondria in 4D, quantitative analysis methods for the resulting datasets have been lacking. Here we present MitoTNT, the first-in-class software for Mitochondrial Temporal Network Tracking in 4D live-cell fluorescence microscopy data. MitoTNT uses spatial proximity and network topology to compute an optimal tracking assignment. To validate the accuracy of tracking, we created a reaction-diffusion simulation to model mitochondrial network motion and remodeling events. We found that our tracking is >90% accurate for ground-truth simulations and agrees well with published motility results for experimental data. We used MitoTNT to quantify 4D mitochondrial networks from human induced pluripotent stem cells. First, we characterized sub-fragment motility and analyzed network branch motion patterns. We revealed that the skeleton node motion is correlated along branch nodes and is uncorrelated in time. Second, we identified fission and fusion events with high spatiotemporal resolution. We found that mitochondrial skeleton nodes near the fission/fusion sites move nearly twice as fast as random skeleton nodes and that microtubules play a role in mediating selective fission/fusion. Finally, we developed graph-based transport simulations that model how material would distribute on experimentally measured mitochondrial temporal networks. We showed that pharmacological perturbations increase network reachability but decrease network resilience through a combination of altered mitochondrial fission/fusion dynamics and motility. MitoTNT’s easy-to-use tracking module, interactive 4D visualization capability, and powerful post-tracking analyses aim at making temporal network tracking accessible to the wider mitochondria research community.

## Author summary

Mitochondria are the powerhouses of the cell and assume critical roles in cell fate, cell signaling, and cellular health. Mitochondria dysfunctions underlie many human diseases, including cancer, diabetes, cardiovascular and neurodegenerative diseases. Contrary to the bean-shaped textbook depiction, mitochondria form large interconnected and dynamic network structures in cells. These four-dimensional networks (space and time) could only recently be captured through advanced imaging methods such as lattice light-sheet microscopy (LLSM). The current challenge has been the lack of analysis tools to extract quantitative information from the enormous LLSM datasets. Here, we present the software MitoTNT (Mitochondrial Temporal Network Tracking) to solve this data analysis problem. MitoTNT uses the distance and topology information in the mitochondrial network to track the mitochondria over time. MitoTNT achieves >90% tracking accuracy in synthetic ground-truth datasets and agrees well with previously published motility values from experimental data. Using MitoTNT, we uncovered distinct network movement patterns, fission and fusion preferences, and network-level responses to drugs. To make temporal mitochondrial network tracking widely accessible, MitoTNT comes with an easy-to-use tracking module, an interactive 4D visualization capability, and powerful post-tracking analyses.

This is a *PLOS Computational Biology* Methods paper.

## Introduction

Mitochondria are membrane-bound organelles in cells that provide up to 90% of the cellular energy and are thus fundamental to almost all processes of life from inheriting genetic information to retaining molecular order [1,2]. In mitochondrial diseases, the function of mitochondria is impacted, leading to diminished energy production and cell and organ dysfunction. This is particularly true in high-energy demand organs such as the muscles, heart, and brain. A vast array of diseases such as metabolic disorders [3], developmental disabilities [4], epilepsy [5], neurodegenerative disease [6–8], cancer [9], and aging [10,11] may result from mitochondrial dysfunction. Progress in developing pharmacological modulation of mitochondria has been limited, potentially due to the current difficulty in quantitatively measuring the behavior of the cellular mitochondrial network with sufficient spatial and temporal details.

Measuring the dynamic mitochondrial network is challenging. Far from the solitary kidney bean shapes depicted in many textbooks, interconnected somatic mitochondrial tubules fill all three spatial dimensions and undergo continuous changes in the fourth dimension of time through active and passive motion, fission, and fusion. Conventional fluorescence microscopy technology has been inadequate to simultaneously capture the full spectrum of both mitochondrial morphology and dynamics in all four dimensions (4D). The advent of high-framerate low-phototoxicity fluorescence microscopes such as lattice light-sheet microscopy (LLSM) [12,13] has now made the detailed 4D characterization of temporal mitochondrial networks possible. However, quantitative analysis of this data remains a challenge.

The majority of existing quantitative analysis software was designed for two-dimensional (2D) fluorescence images of mitochondria (MyToe [14], MitoSPT [15], QuoVadoPro [16]). For three-dimensional (3D) fluorescence images, MitoGraph [17–19] is a tool for the segmentation and quantitation of 3D mitochondrial network morphology yet lacks temporal analysis. The software packages TrackMate [20] and Mitometer [21] can operate on 4D time-lapse fluorescence microscopy data by performing center-of-mass tracking. However, the abstraction of every mitochondrial fragment as a single object poses inherent limitations for segmenting network structure and investigating sub-fragment level information.

Here we present MitoTNT, the first-in-class software for the tracking of the 4D mitochondrial network. MitoTNT is written in Python and builds on the established tools MitoGraph for segmentation and ChimeraX [22,23] for intuitive visualization. Mitochondria tracking is achieved by solving a linear assignment problem (LAP) that utilizes both spatial and network topology information. Tracking precision was validated both in-silico and in-vitro. A reaction-diffusion simulation of the mitochondrial network was created to provide in-silico ground truth for testing. In-vitro data of mitochondrial networks was created using LLSM in human induced pluripotent stem cells (hiPSCs), and other cell types (HEK293 cells and neural progenitor cells). We demonstrate that MitoTNT’s high-resolution mitochondrial network tracking is accurate and provides an unprecedented level of detail for mitochondria motility measurement, fission/fusion event detection, and temporal network analysis.

## Results

### Preserved topology enables 4D mitochondrial network tracking

**Fig 1:**
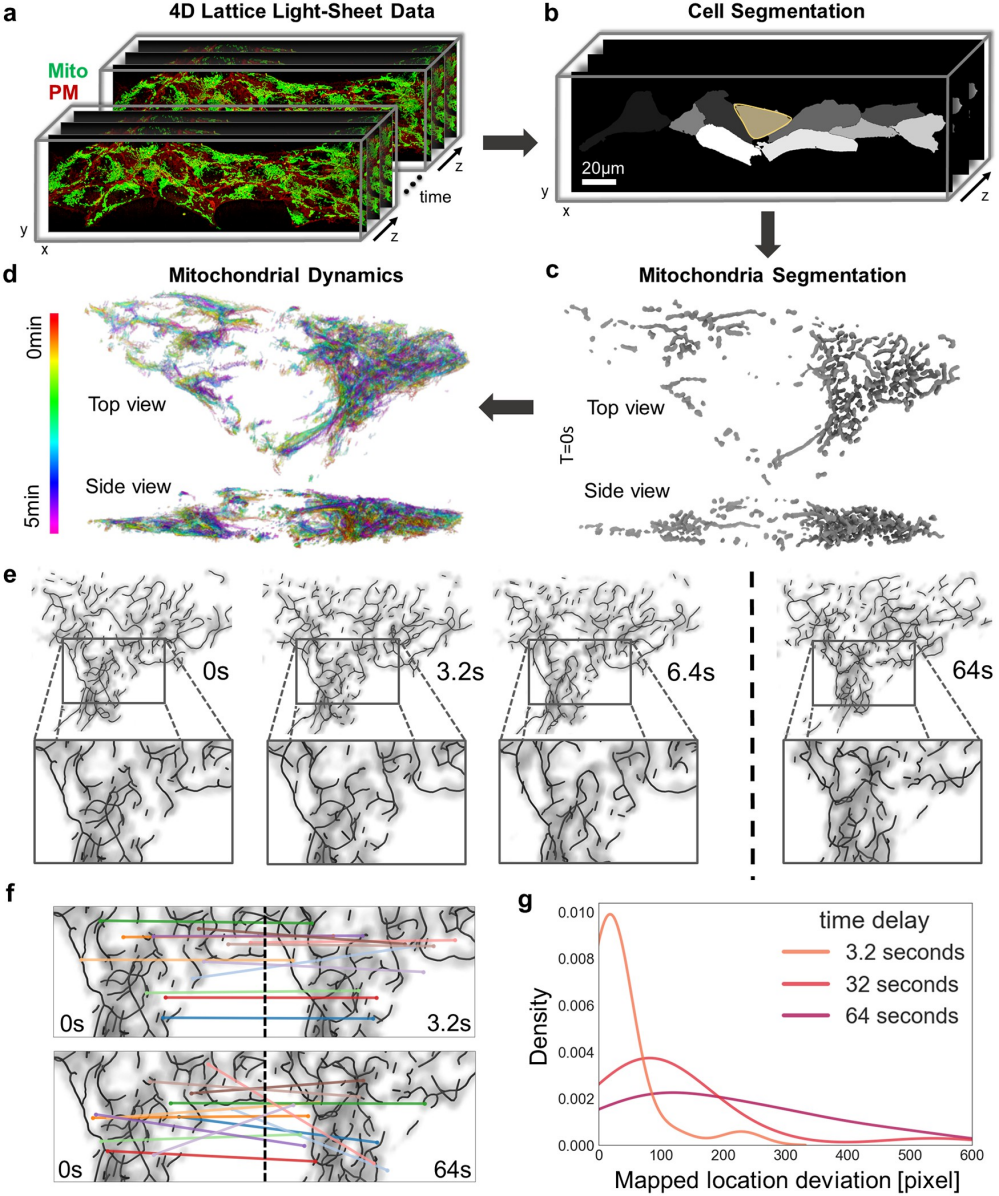
Mitochondrial network topology is preserved in high-framerate 4D fluorescence microscopy data. **a**, Representative 4D (3D+time) lattice light-sheet microscopy data of a hiPSC colony labeled with MitoTracker (mitochondria, green) and expressing CAAX-RFP (plasma membrane, red). **b**, Individual cells in the colony are segmented based on the plasma membrane marker. **c**, Mitochondria fluorescence signal in a single cell is segmented using MitoGraph. **d**, Mitochondrial network skeleton dynamics over 5 min every 6.4s (time red to purple). **e**, Mitochondrial fluorescence density and segmented network skeleton are overlaid and shown for frame numbers 0, 1, 2, 20 at frame interval 3.2s. **f**, Scale-invariant feature transform (SIFT) maps image features for two frames separated by 3.2s (top), and 64s (bottom). The image size for the mapped region is 1034px by 642px. **g**, Pixel deviation between SIFT-mapped feature locations at different time intervals.

Our first aim was to confirm that high-framerate fluorescence imaging of the 4D mitochondrial network retains enough information for reliable tracking. The somatic mitochondrial networks of tall cuboid hiPSCs were used as a model system (Fig 1a). LLSM was used to acquire imaging volumes at 3.2s per volume. After deskewing and deconvolution, individual cells were computationally segmented based on the plasma membrane signal (Fig 1b). MitoGraph was then used to segment the mitochondrial network for consecutive imaging volumes (Fig 1c and 1d). At a 3.2s frame interval, we observed that changes of the 4D mitochondrial network are predominantly limited to small movements and remodeling events while the overall network structure appeared to be conserved from frame to frame (Fig 1e). We then quantified this conservation at several acquisition frame rates by applying the scale-invariant feature transform (SIFT) [24]. For small time intervals, SIFT was able to correctly assign network features between frames (Fig 1f top), but failed for longer time intervals (Fig 1f bottom). We found that at high volumetric frame rates, mitochondrial network topology is preserved (Fig 1g). In the next section, we aim to use this conserved temporal information to achieve 4D mitochondrial network tracking.

### 4D mitochondrial network tracking using spatial and topological information

**Fig 2:**
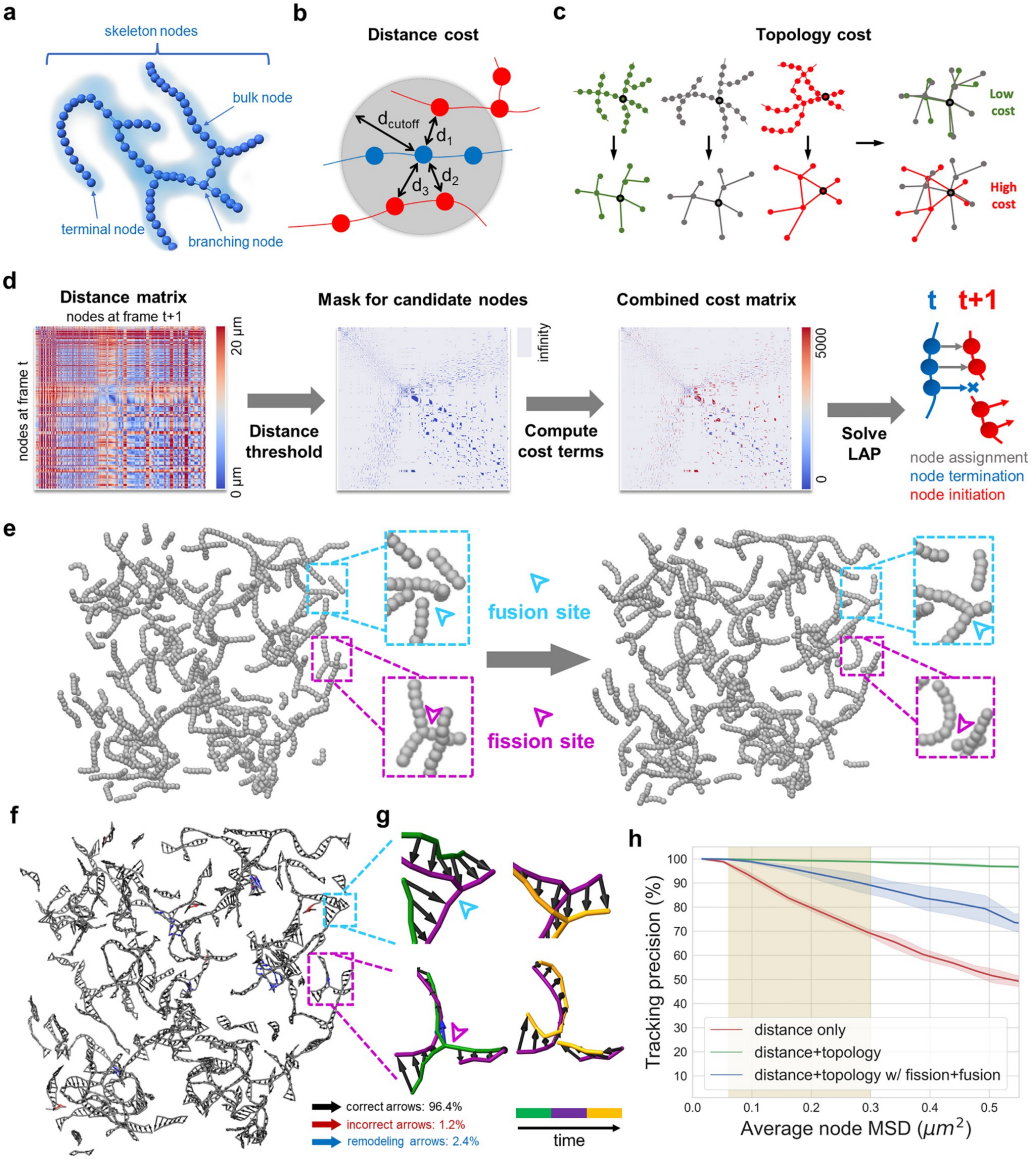
Algorithm design and in-silico validation of 4D mitochondrial network tracking. **a**, Discretized nodes along the segmented mitochondria skeleton serve as the basis for network tracking. Terminal, branching, and bulk nodes are treated equally and termed skeleton nodes. Cloud: fluorescence density; sphere: skeleton node. **b-c**, Cost terms used for the linear assignment problem (LAP) formulation of node tracking. Spatial proximity is measured as distances between nodes within two consecutive frames. Topology cost is computed using a graph comparison that assigns low cost for similar local topology. **d**, LAP formulation of node tracking for the mitochondrial network. From left to right: 1) pairwise distance matrix for nodes at frames T and T+1; 2) thresholds to eliminate nodes too far to be tracked; 3) spatial separation and network topology constraints; 4) the solution to the LAP yields the tracking results as linked node pairs, along with terminated and initiated nodes. **e**, Two consecutive frames of a reaction-diffusion mitochondrial network simulation with representative fusion (cyan) and fission (magenta) positions pointed out by the arrows. **f**, Temporal network tracking for the simulated mitochondria for two consecutive timepoints (black: correct arrow, red: incorrect arrows, blue: incorrect arrows at the fusion/fission sites). **g**, Magnification of the example in-silico fusion (cyan) and fission (magenta) events in e). **h**, The y-axis denotes tracking precision which is the percentage of nodes that are correctly tracked (based on simulation ground-truth). The x-axis denotes average node mean squared displacement (MSD) per frame, which is linearly proportional to the frame interval according to *MSD* = 6*Dτ*. MSD is computed by converting simulation units to real world units so that it can be comparable to experimental data. Tracking is then performed for three scenarios: 1) red: tracking on simulations without fusion/fission using distance cost only (similar curve for simulation with fusion/fission); 2) green: tracking on simulation without fusion/fission using distance and topology costs; 3) blue: tracking on simulation with fusion/fission using distance and topology costs.

Conventional graph representation of mitochondrial networks represents each branch/segment as an edge between either terminal (degree = 1) or branching (degree>2) nodes [17–19]. To achieve full-resolution network tracking, we include bulk (degree = 2) nodes [25] equally spaced along the skeleton between terminal and branching nodes at the resolution of the fluorescence data. We use the term “skeleton nodes” to refer to all three types of mitochondrial nodes (Fig 2a). The discretization of the mitochondrial fluorescence data into such pixel-based skeleton nodes can be automatically performed by the software MitoGraph. We used the skeleton nodes as the fundamental units for tracking.

We then formulated 4D mitochondrial temporal network tracking as an optimization problem that uses information preserved between consecutive frames. We found that spatial proximity (Fig 2b) and network topology (Fig 2c) are unique features that facilitate temporal tracking. At high frame rates, mitochondrial motion is limited, and the nodes located close to the current position in the next frame tend to be the correct candidates. However, this distance metric quickly decorrelates in dense network regions. Similarly, the mitochondrial network topology remains relatively stable at high frame rates and only decorrelates at high fission/fusion rates of the network. We developed a topological dissimilarity score to capture this parameter. The score is computed using a fast alignment-based graph comparison method (see Methods) to measure how different the network topologies around any two candidate nodes are. Nodes embedded in a similar local network topology are more likely to be linked in time.

Similar to established particle/object tracking methods [20,21,26], we formulated the network tracking problem as a linear assignment problem (LAP) that solves for the optimal node assignment through constraints (Fig 2d). First, the distances between nodes in two consecutive frames T, T+1 were computed as a pairwise distance matrix. Next, local distance thresholds were estimated for each node at frame T. Nodes located within these thresholds at frame T+1 were considered candidate nodes while those beyond were ignored. Then, network topology was incorporated using the topological dissimilarity score for each candidate node pair (node at T and candidate node at T+1). The distance and topology costs were then combined with the optimal weights (see S3 Fig). Mitochondrial dynamics and imaging artifacts often contribute to fluctuations in the number of skeleton nodes. To account for this fluctuation, we added additional auxiliary matrices, thereby permitting three options for a temporal assignment: 1) link two nodes between frames, 2) terminate a node in the current frame, or 3) initiate a new node in the next frame. Finally, the frame-to-frame tracking result is given as the optimal node assignment to the LAP by minimizing the global sum of the final cost matrix. Gap closing is performed at the end of frame-to-frame tracking in order to connect prematurely terminated node tracks, using the same cost terms (see Methods).

### In-silico validation of MitoTNT through spatial reaction-diffusion simulations of mitochondrial networks

Our next aim was to validate our tracking algorithm using synthetic data as ground-truth. A meso-scale reaction-diffusion simulation was developed to model temporal mitochondrial networks (Fig 2e). We used the ReaDDy [27,28] framework to model mitochondria as connected mitochondrial skeleton particles that were held together by bond, angle, and repulsion potentials (S5a Fig). Mitochondrial motion was assumed to be diffusive. Fission (Fig 2e, magenta) and fusion (Fig 2e, cyan) were included as structural reactions such that fission reactions remove a bond between skeleton nodes and fusion reactions create a bond between unbound skeleton nodes (S5b Fig). The spatial distribution and density of the in-silico mitochondrial network was modeled based on the imaged mitochondrial networks. The experimental networks were found to resemble a mixture of Erdös–Rényi random networks (S5c and S5d Fig). Experimental observations of fission and fusion rates were adjusted through iterative sampling of fission and fusion reaction rates.

Tracking accuracy of our algorithm was subsequently tested using this simulation as ground-truth. We found that each fragment of the mitochondrial network, as well as fission and fusion events are tracked faithfully with few mis-assignments (See Fig 2f and 2g), where correct arrows are colored in black, incorrect arrows in red, and incorrect arrows due to network remodeling in blue. We found that the distance constraint alone results in relatively poor tracking performance (Fig 2h, red curve) likely due to ambiguous assignments in the dense mitochondrial network. In contrast, when paired with the topology constraint, consistently reliable tracking was achieved with fission and fusion switched off (>98% precision, Fig 2h green) or on (> 90% precision, Fig 2h blue) in the regime relevant for LLSM (shaded region, see Methods).

### In-vitro validation and evaluation of 4D mitochondrial network tracking

**Fig 3:**
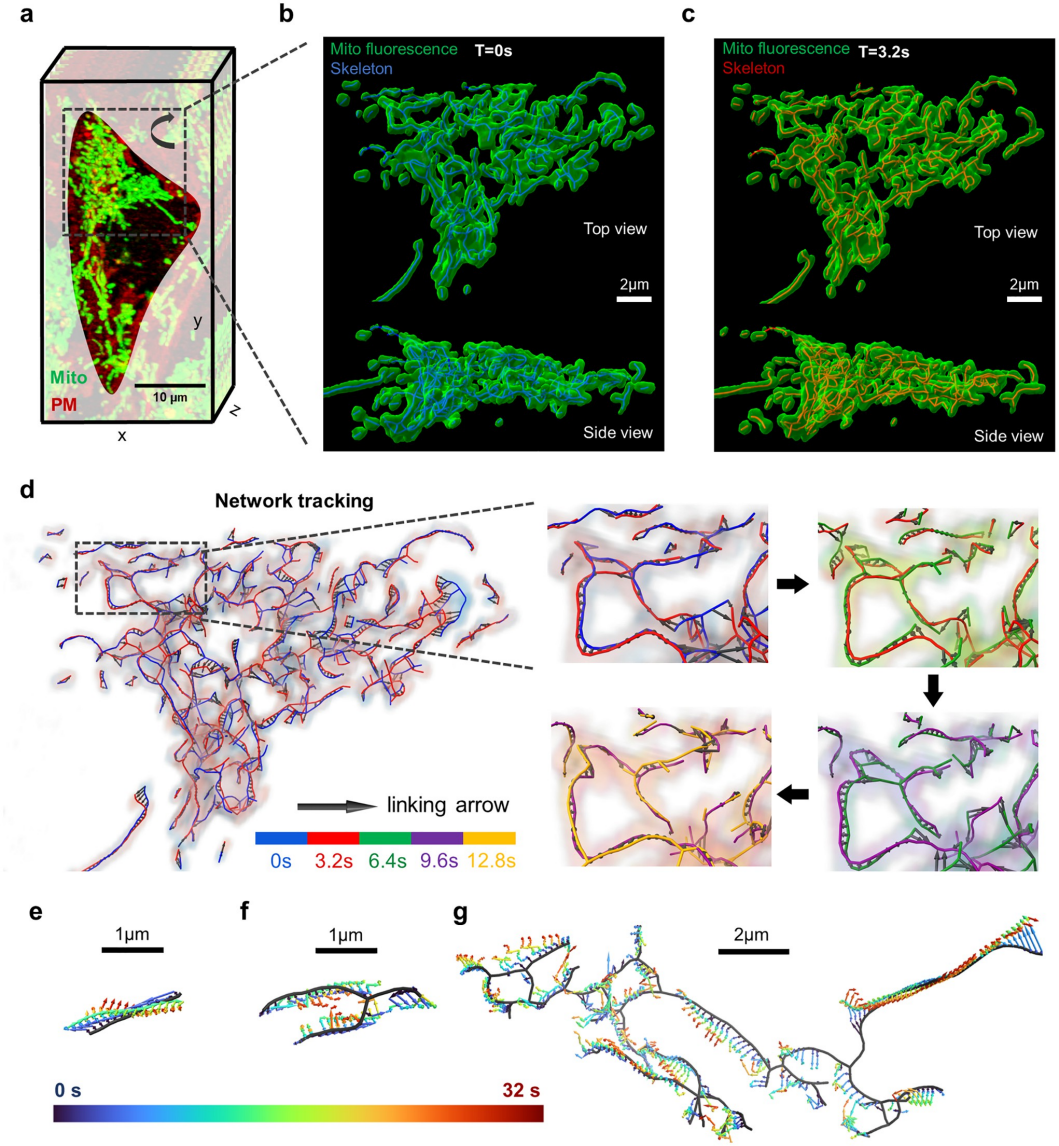
In-vitro validation and evaluation of 4D mitochondrial network tracking. **a**, LLSM volumetric snapshot of a segmented cell. Green: mitochondrial network. Red: plasma membrane. **b-c,** Fluorescence signal and segmented network skeleton are overlaid for two consecutive frames (blue: 0s, red: 3.2s). **d**, Left: tracking of the skeleton nodes for the two frames in b) and c) are visualized by black arrows. Right: zoom-in to a representative region tracked over 12.8s. The skeletons are colored in blue, red, green, purple and orange in the order of time. **e-g,** Tracking of three representative mitochondrial network fragments for 32 seconds (time blue to red). e) A small fragment displays twisting motion. f) A medium-size fragment displays inward motion. g) A large fragment displays complex motion patterns.

We next validated our tracking algorithm on LLSM data of 4D mitochondrial networks in cultured cells. CAAX-RFP hiPSC colonies were labeled with MitoTracker Green and imaged at 3.2 seconds per volumetric frame for a duration of 5 minutes. Single-cell mitochondrial network were segmented using MitoGraph (Fig 3a–3c). We then used MitoTNT to track mitochondrial motion. Branch movements were well captured by the tracking arrows, despite occasional topology variations between frames. (Fig 3d and S6 Fig). We observed individual fragments displaying a wide range of movement patterns that include translational, and rotational components. Branches of the same mitochondrial fragment can simultaneously undergo motions with different orientations and modes. Here we showcased three examples: 1) a small fragment exhibiting twisting motion (Fig 3e), 2) a medium-sized fragment exhibiting concentric inward motion (Fig 3f), and 3) a large fragment exhibiting a convolution of different motility patterns (Fig 3g). To test the generality of MitoTNT, we also imaged and tracked HEK293 cells (an immortalized cell line), and neural progenitor cells (a cell type differentiated from hiPSCs). In both cases, MitoTNT managed to generate high-fidelity mitochondrial network tracking (S7 Fig). To further test the generalizability of MitoTNT, we investigated modes of mitochondrial dynamics that are different from fission and fusion. We tested the tracking of branch extension [29] and toroid formation [30] events in mitochondrial networks and found that MitoTNT is able to track both modes well (S9 Fig).

### High-resolution mitochondria tracking reveals heterogeneous sub-fragment motility and correlated movement patterns

**Fig 4:**
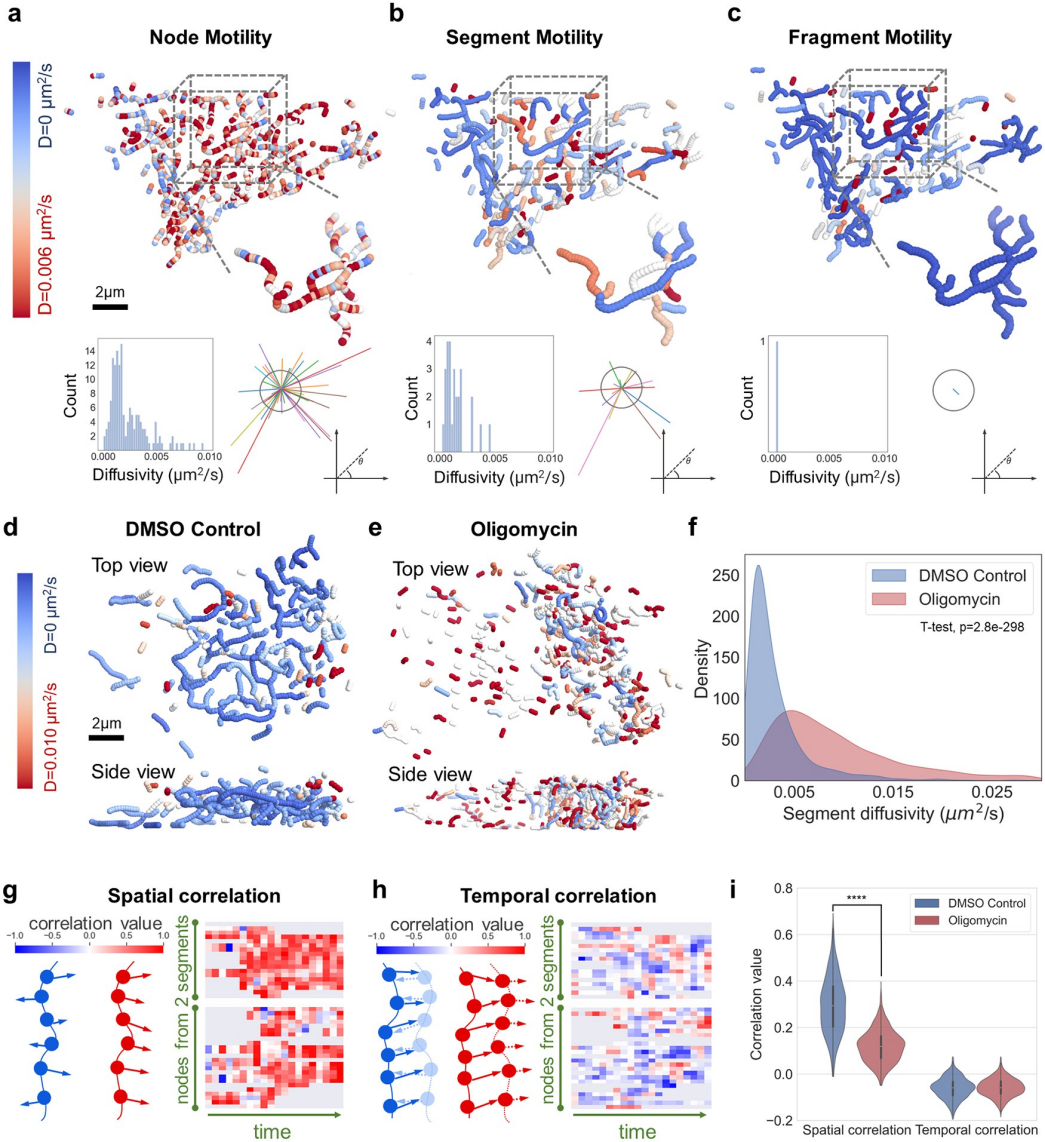
Mitochondrial network motility analysis. **a-c**, Top: Mitochondrial nodes are colored by diffusivity at node, segment, or fragment levels from high (red) to low (blue) diffusivity. Bottom left: distribution of diffusivity values, bottom right: linking vectors compared to a fixed reference vector. **d-e**, Spatial structure of somatic mitochondrial network colored by mitochondria segment diffusivity in control and oligomycin-treated cells. **f**, Kernel-smoothed distribution of segment diffusivity for 2552 segments in control cells (blue), and 2376 segments in oligomycin-treated cells (red). T-test is performed with p-value = 2.8e-298. **g**, Spatial tracking vector correlations between neighboring nodes. Left: illustration. Right: heatmap of vector correlation for segment nodes (columns) at different timepoints (rows). **h**, Temporal tracking vector correlation for the same node at consecutive frames. Left: illustration. Right: heatmap of correlation values for segment nodes (columns) at different timepoints (rows). **i**, Violin plot of the spatial and temporal correlation values between control (blue) and oligomycin-treated (red) cells.

Depending on the level of granularity required for the biological question of interest, tracks for the skeleton nodes (Fig 4a) that belong to the same segment (Fig 4b), or the same fragment (Fig 4c) can be computed and mapped on the network structure. We found that somatic mitochondrial motility is diffusive not only on the fragment-level but also on the mitochondrial skeleton node-level (S10 Fig). We observed that the high-resolution tracking on the level of mitochondrial skeleton nodes illustrates that mitochondrial motility and dynamics exhibit complex spatial and temporal details and a heterogeneity in speed and orientation (Fig 4a–4c, lower panel). Finally, we compared our high-resolution tracking results to previously published values of lower-resolution center-of-mass tracking [21]. We found that our average mitochondrial network fragment motility for hiPSC mitochondria (0.06±0.03 μm/s) is in good agreement with motility data from 3D spheroids (0.03 μm/s) and 2D adherent cells (0.08 μm/s) (S10a and S10b Fig). To investigate whether disruptions of normal mitochondrial function can manifest in network branch motility, we treated the cells with ATP synthase inhibitor oligomycin. We observed that the oligomycin-treated mitochondria are more fragmented and that the resulting small fragments expectedly move considerably faster compared to control (Fig 4d–4f).

To further investigate network branch motility, we correlated tracking vectors between adjacent nodes on the same segment (spatial), and between the same node at consecutive frames (temporal). We observed that spatial correlation along the segment skeleton nodes is predominantly positive (Fig 4g), demonstrating a concerted motion. In contrast, temporal correlation between frames is predominantly zero (random motion) to slightly negative (oscillating motion), while interspersed with short period of positive values (directional motion) (Fig 4h). Statistics on many mitochondrial segments confirmed that the mitochondrial branches move as a unit but in a relatively random manner (Fig 4i, temporal correlation). We observed that while temporal motion correlation remains similar, the spatial motion correlation is reduced (Fig 4i, spatial correlation) after oligomycin treatment.

### Mitochondrial network tracking reveals local fission and fusion fingerprints and asymmetric fission and fusion preferences

**Fig 5:**
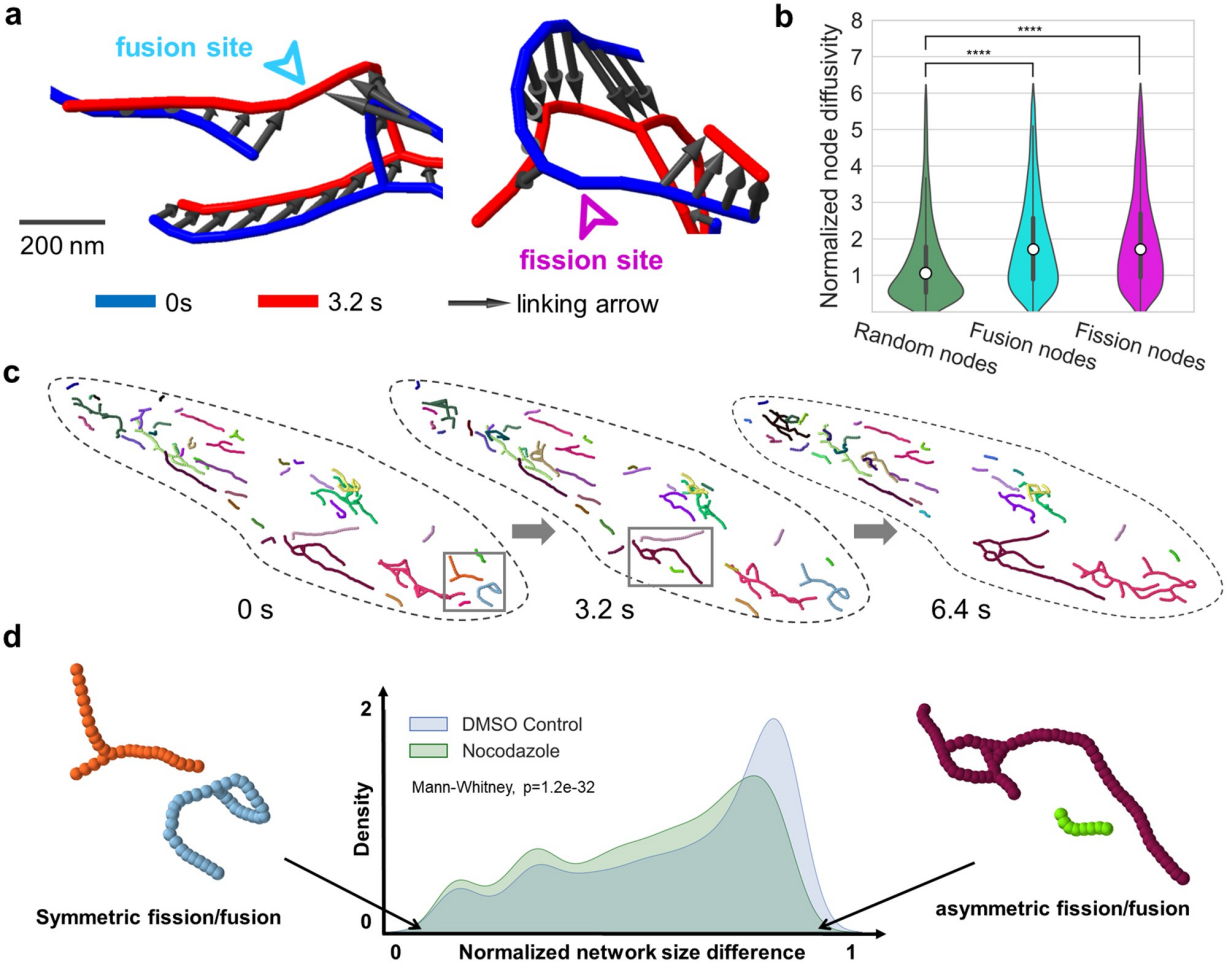
Mitochondrial network remodeling analysis. **a**, Representative snapshots of a tracked fusion event (left), and fission event (right). **b**, Node diffusivity values are significantly lower for randomly selected nodes (green) as compared to nodes undergoing fusion (cyan) and fission (magenta). Student’s t-test are applied, and p-values are 6.565e-25 and 1.237e-24 for random vs. fusion nodes and random vs. fission nodes, respectively. **c**, Representative tracking of mitochondrial fragments in hiPSCs over three timepoints where each fragment is uniquely colored. **d**, Analysis of fission/fusion preferences with respect to fragment size for control (blue) and nocodazole-treated condition (green). Normalized network size difference measures the differences in # nodes for the fragments undergoing remodeling events, normalized by # nodes of the larger fragment. Mann-Whitney test is used to test whether the underlying distribution is the same, with p-values = 1.2e-32.

Our high-resolution network tracking allows us to precisely locate fission and fusion events in the mitochondrial network with sub-fragment spatial resolution and high temporal fidelity. To provide mechanistic insights into network remodeling, we compared the motility between randomly selected nodes and nodes undergoing fission and fusion. We observed that the mean normalized diffusivity for nodes undergoing fission and fusion is nearly two times the diffusivity for randomly chosen nodes (Fig 5b). This data suggests that mitochondrial fission and fusion remodeling might involve local rearrangements at the event site as suggested previously [31].

Based on node tracking, each individual mitochondrial network fragment can be tracked (Fig 5c) and the selectivity of fission and fusion events recorded in terms of fragment size. For each fission or fusion event, the normalized network fragment size difference was computed, with values close to 0 corresponding to a symmetric fission/fusion (Fig 5d, left), and values close to 1 corresponding to an asymmetric fission/fusion (between large and small fragments) (Fig 5d, right). We found that there is a significant portion of asymmetric fission/fusion events (Fig 5d, blue). Asymmetric fission/fusion events between large healthy mitochondria and small unhealthy mitochondria have been proposed to separate dysfunctional mitochondria targeted for mitophagy, or rescue damaged mitochondria by supplying essential materials [32,33]. We hypothesized that this dynamic selectivity bias is facilitated by cytoskeleton such as microtubules. In cells treated with 10 μM of nocodazole to disrupt microtubules, we observed a decrease in asymmetric fission/fusion (Fig 5d, green). This observation points to a potential role of microtubules in mediating selective fission/fusion.

### 4D mitochondrial network tracking shows drug-dependent network remodeling rates, network transport, and network resilience

**Fig 6:**
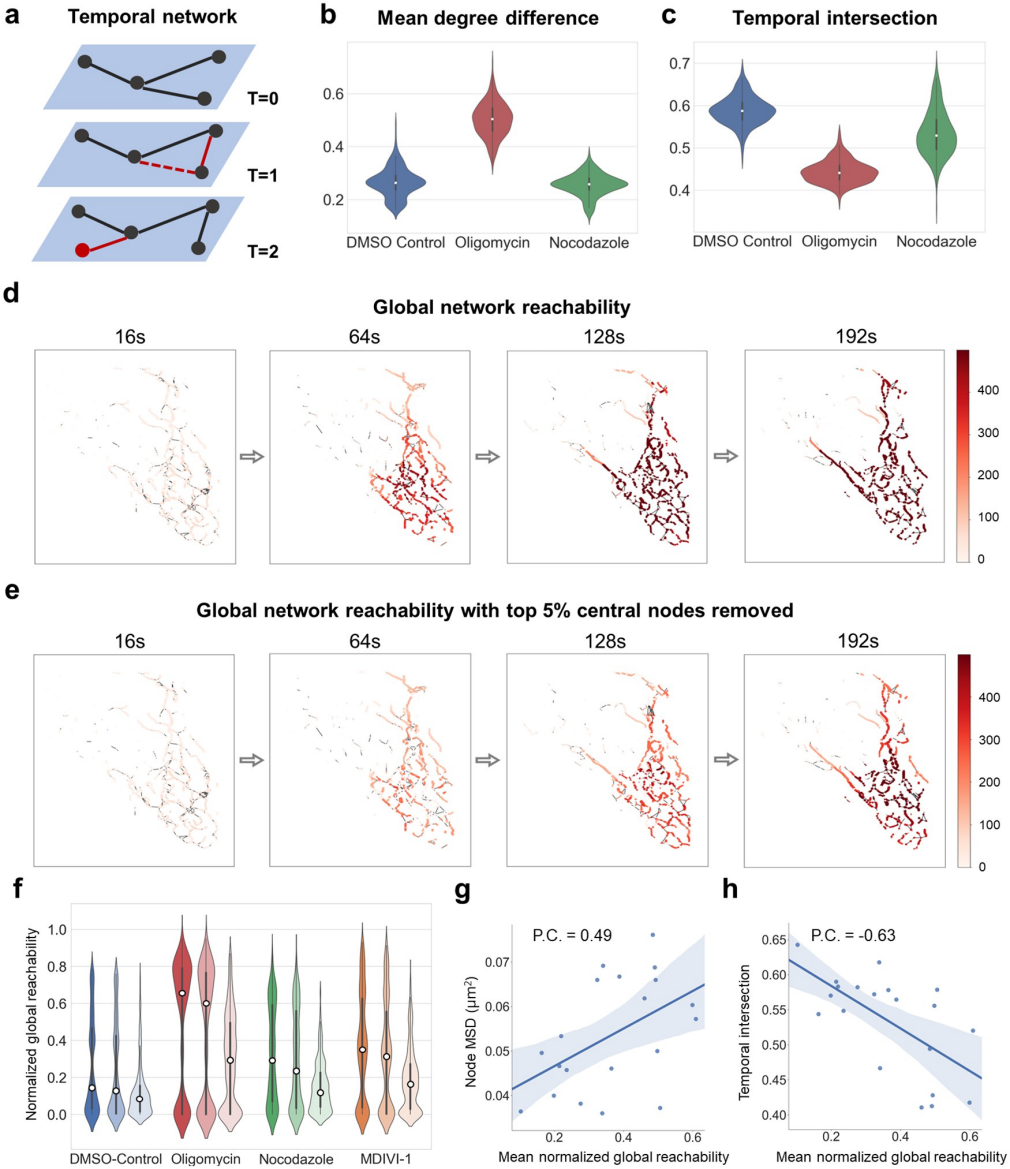
Temporal characteristics of mitochondrial network remodeling, flux, and damage resilience. **a**, Temporal networks display node and edge dynamics that have an influence on network transport and resilience (newly added or removed nodes/edges highlighted in red). **b**, Mean degree difference between control, oligomycin, and nocodazole. **c**, Temporal intersection between control, oligomycin, and nocodazole. **d**, Global network reachability in a representative somatic mitochondrial network depicted as a color gradient (dark: high reachability, light: low reachability). **e**, Global network reachability where the top 5% highest betweenness-centrality nodes were removed. **f**, Mean normalized global reachability in different drug conditions. The three violin plots for each condition correspond to no nodes removed (left), 5% random nodes removed (middle), and 5% most connected nodes removed (right). **g**,**h**, Correlation of network reachability with node MSD and temporal intersection.

4D mitochondrial network tracking allowed us to investigate the mitochondrial network from the perspective of a temporal network (Fig 6a). Specifically, it is now possible to quantify a) remodeling of the mitochondrial network, b) flux across the network as it moves spatially and is being remodeled, and c) resilience of the network to damage.

To quantify mitochondrial network remodeling, the mean degree difference and the temporal intersection were calculated (see Methods). Low mean degree difference suggests that few nodes break off from the network and reconnect with other nodes. Conversely, low temporal intersection suggests that the network is highly dynamic and does undergo drastic remodeling. We found that the control hiPSCs showed a low mean degree difference of 0.28 (Fig 6b) and a high temporal intersection of 0.58 (Fig 6c), indicating that the network is relatively static with little turnover. In contrast, we observed a 0.52 mean degree difference and 0.44 temporal intersection reflecting a high level of network remodeling for cells under oligomycin treatment. However, treatment with nocodazole did not induce drastic change in neither metric for network remodeling (Fig 6b and 6c).

To quantify transport across the 4D mitochondrial network, we simulated a random walk on the tracked temporal mitochondrial networks and measured the process in the form of network reachability (see Methods). Reachability for a node indicates how easily material/information can reach this node from various parts of the overall network, via the time-respecting paths defined by the network tracking. In control conditions, we observed that almost every part of the network was in reach within ~120s (Fig 6d) and that network nodes showed a low average reachability of 0.18 (Fig 6f). Comparatively higher reachability was reached with nocodazole (0.29), mdivi-1 (a fission protein inhibitor) (0.35), and in particular with oligomycin (0.64).

To quantify mitochondrial network resilience, mitochondrial node reachability was calculated in networks where the top 5% of highest connected nodes were removed, as measured by betweenness of centrality. We observed that the global reachability for a large number of nodes was significantly reduced, particularly those isolated from the larger well-connected fragments. This observation suggests certain central nodes may be essential to the material and information transport within the cellular mitochondrial network (Fig 6f). To quantify the relationship between network motility, remodeling, and reachability, we calculated the Pearson’s correlation coefficients between the mean normalized global reachability, the mean node displacement (Fig 6g), and the node temporal intersection (Fig 6h). The positive correlation with node displacement, and negative correlation with temporal intersection suggests that long-range movements and enhanced network remodeling both lead to quicker percolation through the network.

## Discussion

Here we presented MitoTNT, the first-in-class software for mitochondrial temporal network tracking in 4D volumetric fluorescence microscopy data. Recent advances in low phototoxicity volumetric live cell imaging allow fast high-resolution acquisition of the somatic mitochondrial temporal network. Now, MitoTNT allows the automated tracking of this temporal network for the first time. Based on mitochondria skeleton segmentation and discretization through MitoGraph, MitoTNT solves the linking problem of discretized mitochondria skeleton nodes through time. An efficient, alignment-based graph comparison algorithm was used to capture network topology information and pair it with distance constraints for temporal linking. Tracking was validated using both in-silico and in-vitro methods. We created polymer-based spatial mitochondrial simulations that include fission and fusion reactions and are parameterized to reproduce experimental observations to quantify the tracking fidelity of our algorithm. We found that MitoTNT performs with >90% tracking accuracy on these datasets. When comparing tracking performance on experimental in-vitro datasets, with tracking performed by human experts (S8 Fig), we found that MitoTNT tracks the 4D mitochondrial network accurately and reproduces experimental observables such as mitochondrial diffusivity and speed as compared to published values in the literature.

We highlighted three applications of MitoTNT: 1) high-resolution mitochondrial network motility analysis, 2) sub-fragment mitochondrial fission/fusion detection and analysis, and 3) mitochondria temporal network analysis. For motility analysis, we showed that the previously hidden complexity of sub-fragment motility can now be characterized. By coupling network sub-compartment motility with other mitochondrial fluorescence readouts (e.g., membrane potential, reactive oxygen species, mtDNA nucleoid), future studies employing network tracking will have the potential to investigate the functional aspects of mitochondrial motion in cellular physiology. For node-level fission/fusion analysis, we showed that mitochondrial fission and fusion dynamics can be registered at sub-fragment resolution. Compared to fission/fusion detection for object-based tracking, our approach is highly versatile in distinguishing sub-types of mitochondrial remodeling events such as kiss-and-run events, sustained fission/fusion events, intra- and inter-fragment remodeling. We predict that the high spatio-temporal resolution offered through mitochondrial network tracking will become instrumental in studying selective fission/fusion [34] and mitochondrial quality control [35]. For the tracking of mitochondrial networks as temporal networks, we demonstrated that changes in the topology and transport patterns on such temporal networks is indicative of the underlying mitochondrial states. This approach opened up the entire mathematics of temporal networks for future mitochondrial study.

It is worth noting that the reliability of mitochondrial tracking is directly dependent upon the quality of the microscopy data and fluorescence segmentation. MitoTNT has implemented multiple warnings to remind user of potential imaging and segmentation artifacts present in their dataset. Reliability of the tracking can be evaluated with the help of the provided tracking visualization module. Future efforts might use advances in machine learning [36,37] to improve segmentation reliability.

By combining 4D fluorescence imaging, pharmacological perturbations, network tracking, and functional simulation, data-driven cellular metabolic state profiling can be conducted. We hope that MitoTNT’s extendable software design and open-source availability will help to form a community around mitochondria temporal network study for solving challenges in human health.

## Materials and methods

### Human induced pluripotent stem cell (hiPSC) culture

All studies involving hiPSCs were performed under approval from the University of California San Diego IRB/ESCRO committee. WTC hiPSCs expressing the CAAX domain of K-ras tagged with mTagRFP-T were created at the Allen Institute for Cell Science and obtained through the UCB Cell Culture Facility. hiPSC colonies were expanded on dishes coated with growth factor-reduced Matrigel (Corning, 354230) in mTeSR1 (Stemcell Technologies, 85850) containing 1% penicillin/streptomycin (Gibco, 15140122). Colonies were washed with DPBS (Gibco, 14190144) and detached with accutase (Stemcell Technologies, 07920) before plating onto imaging dishes. Cultures were tested routinely for mycoplasma.

### Drug treatments

All drugs were dissolved in DMSO to make a stock solution and diluted in PBS to prepare a 100X working stock before adding to the dish. Cells were treated with oligomycin (20uM, 2 hr), nocodazole (5 uM, 30 min), and mdivi-1 (10 uM, 12 hr) without wash.

### Live cell imaging

CAAX-RFP hiPSCs were stained with 100 nM MitoTracker Green FM (Invitrogen, M7514) for 30 min prior to imaging. Cells were plated onto 25 mm MatTek dishes and imaged in phenol red-free mTeSR1 (Stemcell Technologies, 85850). Cells were kept under 5% CO_2_ and 37 degrees C. For imaging, we used Zeiss Lattice Lightsheet 7 with 10× N.A. 0.4 illumination objective lens and 48× N.A. 1.0 detection objective lens. We acquired images in two channels: green channel with excitation at 488nm and emission at 512nm; red channel with excitation at 561nm and emission at 597nm. For both channels we used 18% laser power and 8ms exposure. The illumination light-sheet was the Sinc3 beam with length 15 μm, thickness 650 nm and no side lobes. The volume size was 2048 x 448 x 57 pixels or 296.94 x 64.96 x 8.12 μm with isotropic pixel size 145 nm after coverglass transformation. Images were saved with bit depth 16 bits. For each region, we imaged 93 frames with frame rate 3.26 s per volume for total 5 min. For LLSM data processing, we used the Lattice Lightsheet 7 Processing Module on ZEN Blue for deconvolution, deskew, cover glass transformation and bleach correction.

### Cell segmentation

The cell segmentation for individual stem cells in a stem cell colony is performed as follows. The red fluorescence signal from CAAX membrane markers is first normalized and smoothed. We then use the 2D filament filter from Allen Cell & Structure Segmenter [38] to threshold the membrane contour for the middle z-section. By dilating and inverting the membrane contour, seed labels for the watershed segmentation in scikit-image [39] are automatically obtained. In order to ensure that each cell has a single seed label positioned near the center of the cell body, the seed labels are manually corrected in ImageJ [40] if necessary. Next, we use the seed labels from the middle z-section to perform 3D watershed segmentation on the entire z-stack. The resulting cell segmentation masks are again manually checked, and used to crop single cells. Since the cell movement was found to be minimal on our timescales of 5 minutes per movie, we applied the segmentation result of the first frame to the entire movie.

### Mitochondria segmentation

We used MitoGraph 3.0 for mitochondria segmentation. The input for MitoGraph was the fluorescence signal of individual cells segmented in the previous step along with default parameters. Adaptive thresholding with block size of 10 pixels was used to calculate a block-dependent local threshold. The output of MitoGraph includes the segmented mitochondrial network and the segmented mitochondrial skeleton which includes network edges and network node attributes including 3D coordinates, fluorescence intensity, and tubular width. A custom python script was created to read MitoGraph outputs, construct the full-resolution mitochondrial networks, and compile the networks with node attributes as a python-igraph [41] object.

The quality of MitoTNT tracking is directly related to the following factors: a) high quality of the MitoGraph segmentation and b) high correlation in mitochondria localizations between consecutive frames. A high quality of MitoGraph segmentation can be achieved by providing MitoGraph with data that has a high signal to noise ratio between mitochondria and background and that has high enough resolution (e.g. diffraction limited or better) to allow the segmentation of individual mitochondrial fragments from each other. A high correlation in mitochondria localizations between consecutive frames means that mitochondrial fragments cannot have moved too far between consecutive frames. In general, this can be achieved by recording the mitochondrial network motility and dynamics at a high enough temporal resolution. We found that recording the network at ~3s per volumetric frame or better results in good tracking outcomes.

We suggest the following approach to evaluating tracking quality:

1. Visually inspect the MitoGraph segmentation quality in an overlay visualization of raw microscope data and segmentation. Segmentation errors have to be addressed in this step.
2. Visually inspect the tracking accuracy of MitoTNT by comparing the linking arrows from one frame to the next using the MitoTNT visualization module.

### Efficient graph comparison using an alignment-based method

The topological dissimilarity cost term compares the local network topologies between the nodes from two frames. This is an inexact graph matching problem with partial node-correspondence since only the two nodes for which the cost is to be computed, termed the target nodes, are known. Existing methods [42] including graphlet-based methods, alignment-based methods, spectral methods, and recent portrait divergence methods are usually designed for large complex networks, are computationally expensive and application specific. Mitochondrial networks usually have much less convoluted connectivity, and thus a small network neighborhood is usually sufficient for our tracking purposes. The proposed graph comparison algorithm needs to prioritize computational efficiency to accommodate the many iterations over network nodes and timepoints for large LLSM datasets. Thus, we have designed a heuristics-driven, alignment-based method that maps the nodes in two subnetworks and computes the topological dissimilarity score as the norm of the differences between two mapped adjacency matrices.

We define two types of networks: 1) the classic networks consisting of terminal (degree = 1) and branching (degree>2) nodes; 2) the full-resolution networks which include additional bulk nodes (degree = 2), equally spaced along the skeleton. We approached network tracking by tracking all the nodes in the full-resolution networks. We formulate the network tracking problem as a linear assignment problem (LAP) for node assignments under certain spatial and topological constraints.

First, in order to efficiently compare the overall network topology around the target nodes, two selected full-resolution networks are converted to the classic networks by eliminating the bulk nodes and directly connecting the terminal and branching nodes. Second, subgraphs up to a user-defined maximum k-level from the target nodes are used, where the k-th level refers to all the nodes whose shortest path to the target nodes have k nodes. For the subgraphs, breadth-first operations starting from the target nodes are performed to 1) convert the graphs to trees by opening loops, and 2) add pseudo-nodes and pseudo-edges of weight zero to ensure that the numbers of nodes at each level are the same. Third, we define a node dissimilarity score between node pairs, and use this score as the cost in the LAP to solve for the optimal node mapping at a given level. We iterate this step for each level until the maximum level is reached. Now that the graph alignment is complete, the topological dissimilarity score between the target nodes can be given as the Euclidean norm of the differences between the two adjacency matrices with distance weights.

### Skeleton node tracking

To track nodes between frames T and T+1, the node coordinates are used to calculate a pairwise distance matrix where element at row m and column n equals the distance between node m at frame T and node n at frame T+1. To limit nodes under consideration for a temporal link, a threshold is implemented. The user can specify the threshold as the distance to the N-th closest neighbor for each node. Additionally, the user can input the maximum node speed, such that nodes located beyond maximum node speed * frame interval are not considered for linking. In our case, we found that the distance to the 10^th^ nearest neighbor is sufficient for tracking at relatively high framerate. The graph comparison score (topology cost) is then computed for the candidate node pairs. The combined cost matrix is the product of distance and topology cost matrices, whose weight can be adjusted by the exponents. We used equal weighting with exponents both equal to 1. We then added the self-assignment matrices with alternative cost equal to the 98^th^ percentile of all previous assignment cost (2 * the minimum of all potential costs for the first frame). The auxiliary matrix is filled with blocking values. The exact definition of self-assignment matrices and auxiliary matrix can be found in the U-track paper [26]. The final cost matrix is then solved using the Jonker-Volgenant algorithm [43]. The tracks of network nodes are then stored and dynamically updated during frame-to-frame tracking.

The tracking arrows based on the global optimal assignment sometimes produce misassignments that can be identified with a few physical constraints:

1. The displacement for each arrow in one segment should be smaller than 3 * the mean displacement for the largest set of arrows in the same segment that are tracked together. This effectively removes unrealistically long arrows by using the other arrows in the segment as the reference.
2. Each tracked node should have at least one other neighboring node that is tracked to the same segment in the next frame. Single node tracked to an isolated segment is very likely a mis-assignment.
3. For all the tracking arrows originated from the same segment, compute the segment average tracking vector. Denote tracking arrows whose angles away from the segment average vector are < 30 degrees as the reference vectors. For each of the other nodes, find the 2 closest nodes around the position of the node displaced by the average of the reference vectors. If the position is not pointed by the reference vectors, reassign the node to the node at this position (in the order of proximity to the predicted position). This constraint is based on the observed concerted mitochondrial segment motion and is effective in correcting crossing arrows between segments at two timepoints.

In case the following thresholds are violated, MitoTNT will trigger warnings to remind users of potential artifacts in experiments or software parameters (both in MitoTNT and MitoGraph). Users can then use the visualization module to evaluate the correctness of tracking and make appropriate decisions when re-evaluating their data.

1. Maximum node speed is the maximum speed each node is allowed to travel. Default to 1 μm/s.
2. Minimum percentage of linked nodes is computed as the number of linked nodes for frames t, t+1 divided by the number of nodes at frame t. This is to ensure that enough nodes can be tracked to produce statistics. Default to 75%.
3. Maximum percentage of node fluctuation is computed as the absolute difference in number of nodes for frames t, t+1, divided by the average number of nodes. This is to respect conservation of mitochondrial mass between consecutive frames. Default to 20%.

### Gap closing

Once frame-to-frame node tracking is complete, node tracks with frames more than min_track_size are qualified for gap closing. The distance and metric costs are computed between the last frame in track A (frame_end_^A^) and the first frame in track B (frame_start_^B^) if both of the following criteria are met: 1<gap size<max_gap_size, where gap size = frame_start_B—frame_end_^A^, distance < gap size * 9 * variance (combined track displacements). Because the total number of tracks can be large (more than 10,000), the cost matrix for all tracks can be memory-consuming, and solving the LAP of such a matrix can be computationally expensive. To solve this problem, we divide the full cost matrix into overlapping blocks to obtain sub-optimal global assignment. First, we sort the tracks based on the start frame so that short tracks that can be linked are positioned relatively close. Next, we crop a NxN block at the top left diagonal of the full cost matrix, where N = max_gap_size * average # of tracks per frame, and use this matrix to close gaps for the tracks involved. Finally, we move this block 0.8*N tracks down the diagonal, to allow gap closing for tracks at the boundary. We repeat this process until the full cost matrix was traversed. This iterative gap closing scheme improves memory and computation performance without noticeably changing the number of closed tracks.

### Mitochondria reaction-diffusion simulation

The particle-based reaction-diffusion simulation tool ReaDDy was used to create a synthetic ground truth dataset of 4D mitochondrial dynamics. We setup a rectangular box potential to represent a cell with dimensions of x, y, z = 20, 20, 5 microns with x, y, z = 2000, 2000, 500 units, where each simulation spatial unit equals 10nm. We added a harmonic boundary potential with force constant k_box_ = 100. Mitochondrial skeleton nodes are represented as individual particles in this box. To form interconnected chains of such particles to represent the mitochondrial network, each node is modeled by the spherical repulsion potential with equilibrium diameter of 350nm, reflecting real-world mitochondrial tubular width [44]. Between each connected node there is a bond potential with k_bond_ = 1, an angle potential with k_angle_ = 10, and equilibrium angle = 180 degrees. All potentials are harmonic.

To generate the initial classic networks, we combine multiple smaller random networks. For each random network partition, n = 60 network nodes are randomly connected with mean degree p = 1.0, and any unconnected nodes are removed. More networks are created until the total number of nodes N = 300. To convert the classic networks into the full-resolution networks in space, we randomly assign the position for one node in the network, and start traversing the entire network starting from that node. Each edge in the classic networks is replaced by 2–5 bulk nodes. Fission events are modeled by removing a bond potential between connected nodes and fusion reactions are modeled by event is modeled by topology dissociation reactions. For each connected component, a random edge is deleted at a probability proportional to the total number of edges. The separated components again can undergo fission events, until there are fewer than four nodes to avoid unrealistically small fragments. Fusion events are modeled by spatial topology association reactions. There exists a certain probability of bond formation when any two nodes are located within two times the node diameter. We designed an iterative simulation scheme to obtain simulations with balanced fission and fusion. First, the system is relaxed and equilibrated for 10^6^ steps without fission/fusion events. Next, initial guesses for the remodeling rates are used to simulate fission/fusion for 2x10^5^ steps. The fission/fusion rates are then updated to compensate for the changes in average fragment size. This cycle is repeated until the changes in average fragment size is small. Lastly, we re-initiate the simulation using the fission/fusion rates established from the latest stable simulation, and adjust the fission/fusion rates every 2x10^5^ steps to compensate any deviation from steady-state dynamics.

To compute the range of MSD accessible to the experimental setup, we used an upper bound of 0.005 μm^2^/s for node diffusivity *D* based on our data and the literature [45], and 1–10 s for frame delay *τ* for LLSM. Thus, using *MSD* = 6*Dτ*, the estimated experimentally relevant MSD range is 0.06–0.3 μm^2^/s. Notice this is assuming the upper bound of mitochondrial motility so the frame rate can be reduced in practice.

### Network motility measurements

To measure the network motility of the mitochondrial network, mean-square-displacements (MSDs) vs. time delay curves are computed following previous works [46,47]. The node ensemble-averaged MSDs are plotted for control and oligomycin (S6a and S6b Fig). The MSDs for single tracks are plotted for control and oligomycin (S6c and S6d Fig). Around 80% of the nodes have a coefficient of determination for linear fit greater than 0.8 (Fig 3f). To map diffusivity onto mitochondrial nodes, we first choose the network nodes at a center frame. Next, we collect the node track coordinates 10 frames before and 10 frames after the center frame. In a last step, we compute the MSDs at various time delays in this time window to determine the diffusivity of each node.

To compute vector correlation, we first obtain the displacement vector for a selected node at a selected frame. Next, we compute the vector correlation between this vector and vectors for the nodes directly connected to it, as well as the vectors for the selected node at timepoints 1 frame before and after the selected frame. The vector correlation is given by the dot product of two vectors, divided by the square of the norm of the longer vector. The final correlation value for the selected node at the selected time is reported as the average of these two values.

### Fission/fusion detection

To detect fission and fusion events in the network, we apply a sliding-window approach to identify nodes that undergo persistent structural changes as opposed to transient segmentation variations. First, the fragment indices for each node are recorded for the *half_win_size* frames before and after the current frame, to form the fragment list. Second, for each network edge, the fragment lists for the connected nodes are compared. Fission will be declared if the fragment lists before the current frame are strictly identical, as well as the fragment lists after the current frame are strictly non-overlapping. Since fusion events can be considered as fission events reversed in time, the opposite criterion is used for fusion detection. In the case that multiple remodeling nodes are located in proximity (less than 5 edges away), the nodes are grouped into a single fission/fusion site. At last, the center of the sliding window is moved to the next frame and the computation is iterated.

### Temporal graph analysis metrics

Temporal intersection is a measure of remodeling of the mitochondrial network over time. It measures how consistent the network remains between timesteps. A higher temporal intersection would mean that the network is more consistent over time, meaning that it has a lower remodeling rate. On the other hand, a lower temporal intersection would mean that the network changes significantly over time, implying a higher remodeling rate. Mathematically, temporal intersection is defined as follows:

$${\left(TI\right)}_{t}=\frac{\left|N_{t}\cup N_{t-1}\right|}{\left|N_{t}\right|}$$

Where (*TI*)_*t*_ is the temporal intersection at timestep t and *N*_*t*_ is the set of nodes at timestep *t*.

Mean degree difference is an alternate measure of how dynamic the network is over time. It describes how the degree of a node changes over time, averaged over all nodes. More specifically, it denotes how dynamic the local connections of nodes are over time. Higher mean degree difference would mean that nodes are connecting and disconnecting more over time, possibly implying higher flux/information exchange. Mathematically, mean degree difference is defined as follows:

$${\left(MDD\right)}_{t}=\frac{\sum_{i}\left|deg\left(n_{t}^{i}\right)-deg(n_{t}^{i-1})\right|}{\left|N_{t}\right|}$$

Where (*MDD*)_*t*_ is the mean degree difference at timestep *t*, $deg\left(n_{t}^{i}\right)$ is the degree of node *n*_*i*_ at timestep *t*, and *N*_*t*_ is the set of nodes at time step *t*.

### Material diffusion simulation on temporal mitochondrial networks

We use a diffusion simulation on temporal dynamic mitochondrial network to understand the flow of material in mitochondrial networks. To calculate global reachability, we initiate a virtual token at every node of the mitochondrial network at the first timestep. Each token is labeled by the source node from which it originated. At every timestep, each node duplicates the tokens it currently has, and transfers them to the connected neighbors. At the end of the simulation, the global reachability for each node is quantified by the number of unique tokens at that node, corresponding to the number of source nodes this node can reach.

## Supporting information

S1 Fig Cell segmentation workflow. a, Fluorescence signal from CAAX membrane marker in the middle plane. b, Cell contour is thresholded. c, Center of the cell is highlighted through dilation and color inversion of the cell contour. This is used as the seed for watershed segmentation algorithm. d, The seed is manually checked and corrected. e, The seed for the middle plane is used to segment cell membrane in 3D using the watershed method.
(TIF)

S2 Fig Illustration for the graph comparison algorithm. a, Visual illustration for the alignment-based graph comparison algorithm. b, Detailed pseudo-code for the algorithm.
(TIFF)

S3 Fig Determination of the optimal cost weighting scheme. a-b, To determine the optimal weighting between distance and topology costs which are combined to form the final cost matrix, we varied the relative weightings and evaluated the tracking precision using the simulation ground-truth. Three exponents (1, 2, 4) in either distance cost a) or topology cost b) are tested while fixing the other cost’s exponent to be 1. Baseline models with either distance only a) or topology only b) are also shown. In all the scenarios, the equal weighting scheme (both exponents equal to 1) consistently demonstrates the highest precision across the full range of MSD.
(TIFF)

S4 Fig Memory-efficient gap closing scheme using overlapping cost matrix blocks. An illustrative gap closing matrix is shown. Row and column indices are node track IDs ranked by the track start frame number. The cost terms are calculated as the product of the distance and topology costs for the end node of the row track, and the start node of the column track. Thus, no assignments are allowed for the lower triangle. Because the number of tracks can be very large, overlapping blocks of the cost matrix are used for track assignments to reduce memory usage and speed up computation. Two track assignments for a track in the overlapped region between two blocks are shown. Because the track is on the edge of the block, the assignment from block 1 has high cost and is sub-optimal (yellow). However, because the block 2 includes more potential tracks, the assignment from block 2 gives the optimal track assignment.
(TIFF)

S5 Fig Network metrics for the mitochondrial simulation. a, Mitochondrial network is modeled by diffusive skeleton particles constrained by angle and bond potentials. b, Fusion and fission events are modeled by random topology changing reactions. c-d, The degree distribution c) and the fragment size distribution d) for the experimental network segmented by MitoGraph (blue dot), and the random network generated for simulation (yellow cross). e, The total number of fragments in the simulation is monitored as a function of simulation timestep to ensure the balance between fusion and fission.
(TIFF)

S6 Fig Extended visualization of MitoTNT tracking in hiPSC. a, Fluorescence intensity over five frames. b, Network tracking for the region in a).
(TIFF)

S7 Fig Mitochondrial segmentation and tracking in HEK293 cell and neural progenitor cell (NPC). a-b, Fluorescence signal, segmented network skeleton, and tracking arrows are overlaid for two cell types other than hiPSC (HEK293 cell which is an immortalized cell line and NPC which is differentiated from hiPSC). One region in each cell type is zoomed in and tracked for 4 frames.
(TIFF)

S8 Fig Tracking validation by comparing MitoTNT outputs to manual tracking. Manual tracking has been done independently by three individuals. One example tracking scenario is shown for manual tracking (top) and MitoTNT tracking (bottom).
(TIF)

S9 Fig Tracking of branch extension and toroid formation modes of mitochondrial dynamics. Segmented network skeleton for two consecutive frames and tracking arrows are plotted for a branch extension event (left) and for a toroid formation event (right).
(TIFF)

S10 Fig Mitochondrial skeleton node diffusivity predominantly follows normal diffusive motion. a, Mean square displacement (MSD) is computed with respect to time delays for skeleton nodes (top) and fragments (bottom). b, Diffusivity values in a) are plotted as a distribution for skeleton nodes (top) and fragments (bottom). c, Node-averaged and time-averaged mean square displacements (blue) are computed with respect to the time delays for two conditions, control and oligomycin. The linear fit line (orange) is shown together with the coefficient of determination (R2) and diffusion coefficient (D). d, Node-averaged MSD for individual tracks in control and oligomycin. e, The goodness of linear fit as measured by R2 is plotted as density distribution, and cumulative distribution function (CDF). R2 close to 1 indicates the data points follow a linear pattern.
(TIFF)

S11 Fig Algorithm for fission/fusion detection based on node tracking. Four tracked nodes are positioned vertically, and seven timepoints are shown horizontally. The center of the sliding window is highlighted with the arrow on top. Three fragments are labeled and colored differently. The fragment indices for each node over time are stored. For each half-window, the index values between every two connected nodes are compared frame by frame. An event is declared if fragment indices in one sliding windows are strictly different in time, while those in the other sliding window are strictly identical in time. This requirement is imposed in order to avoid misidentifying transient segmentation noise as remodeling events.
(TIFF)

S12 Fig Schematics for material diffusion simulation on temporal network. a, Illustration of the global reachability simulation. From each node, material can diffuse into the neighboring nodes or stay in the source node. All possible diffusion pathways over time are marked with an arrow. Time is depicted from left to right. Red, blue, and green arrows indicate representative simulation scenarios. After four timesteps, the target node labeled with X has accumulated three tokens (through the colored transport arrows). b, Illustration of the calculation for temporal betweenness centrality used for determining the central nodes. Each arrow represents the diffusion of material from one node at time t to the next node at time t+1. The temporal betweenness centrality of a node measures how many temporal shortest paths pass through that node. Node Y has a higher number of temporal shortest paths going through it than node X. Thus, node Y has a higher temporal betweenness centrality than node X.
(TIF)
