# Supplementary figures and images for: MitoTNT: Mitochondrial Temporal Network Tracking for 4D live-cell fluorescence microscopy data
Source: PLoS Comput Biol. 2023 Apr 21;19(4):e1011060. doi: 10.1371/journal.pcbi.1011060 (PMC10184899; doi:10.1371/journal.pcbi.1011060)

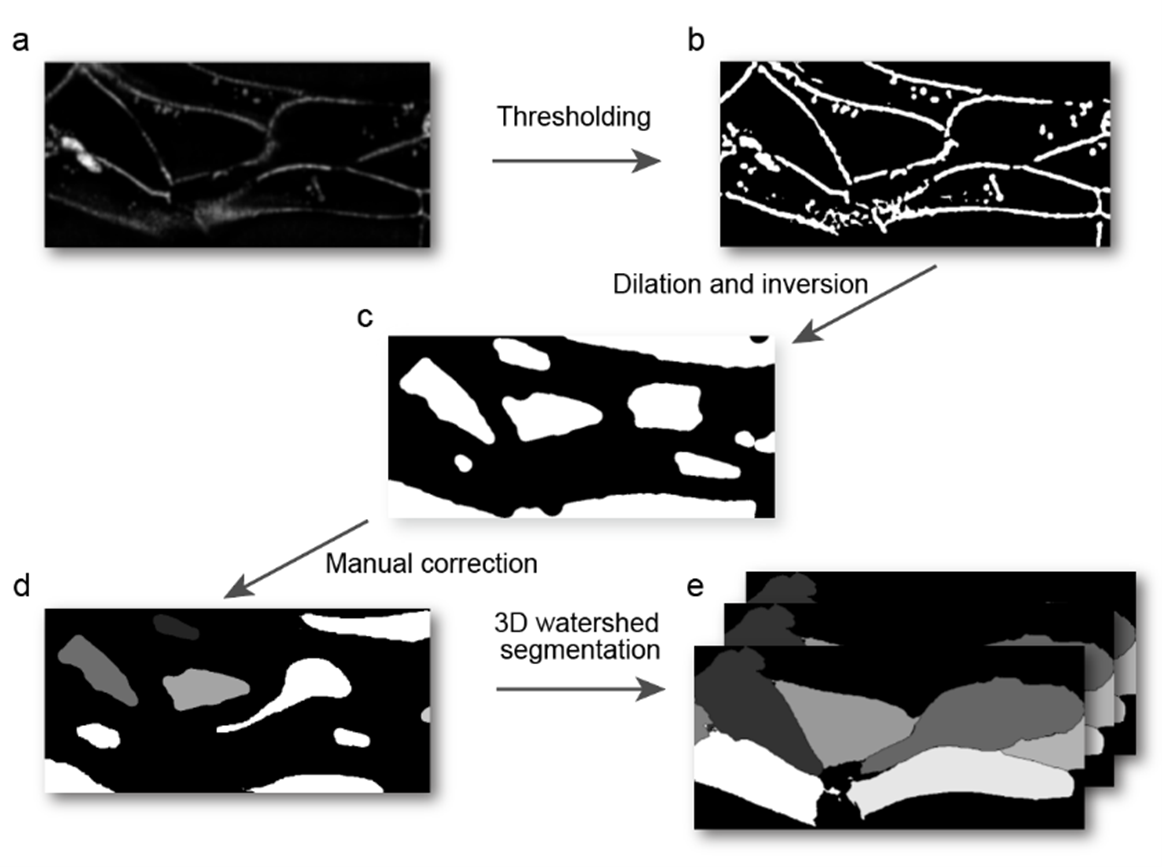

Supplement: S1 Fig — a, Fluorescence signal from CAAX membrane marker in the middle plane. b, Cell contour is thresholded. c, Center of the cell is highlighted through dilation and color inversion of the cell contour. This is used as the seed for watershed segmentation algorithm. d, The seed is manually checked and corrected. e, The seed for the middle plane is used to segment cell membrane in 3D using the watershed method. (TIF) [file pcbi.1011060.s001.tif]

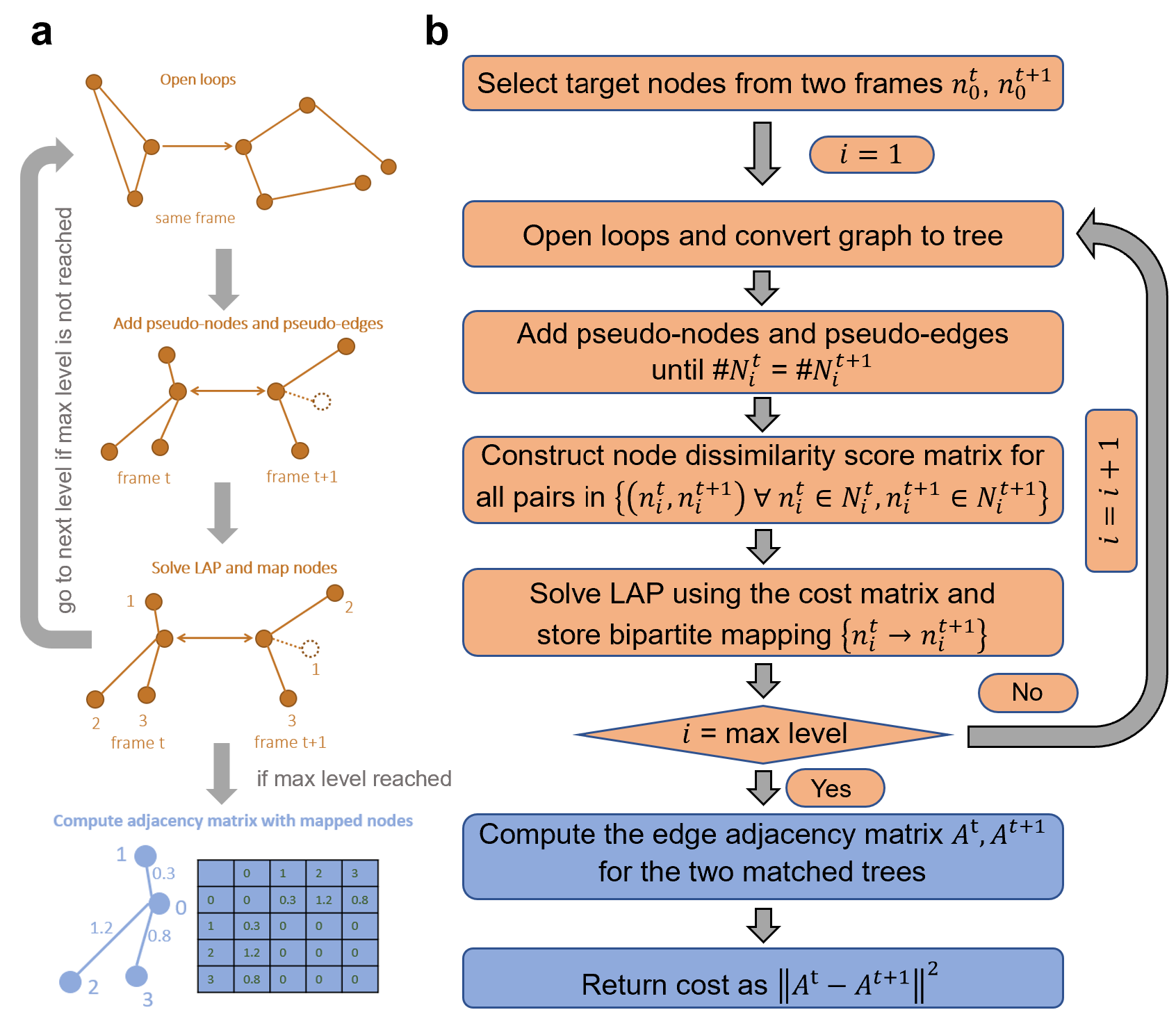

Supplement: S2 Fig — a, Visual illustration for the alignment-based graph comparison algorithm. b, Detailed pseudo-code for the algorithm. (TIFF) [file pcbi.1011060.s002.tiff]

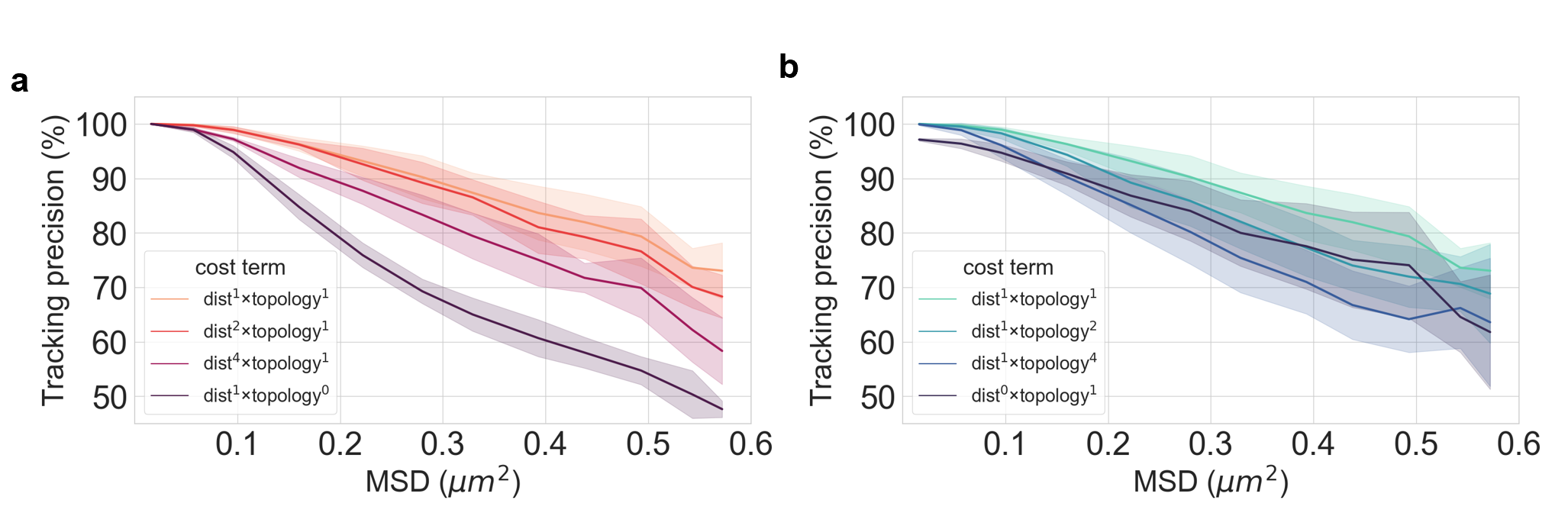

Supplement: S3 Fig — a-b, To determine the optimal weighting between distance and topology costs which are combined to form the final cost matrix, we varied the relative weightings and evaluated the tracking precision using the simulation ground-truth. Three exponents (1, 2, 4) in either distance cost a) or topology cost b) are tested while fixing the other cost’s exponent to be 1. Baseline models with either distance only a) or topology only b) are also shown. In all the scenarios, the equal weighting scheme (both exponents equal to 1) consistently demonstrates the highest precision across the full range of MSD. (TIFF) [file pcbi.1011060.s003.tiff]

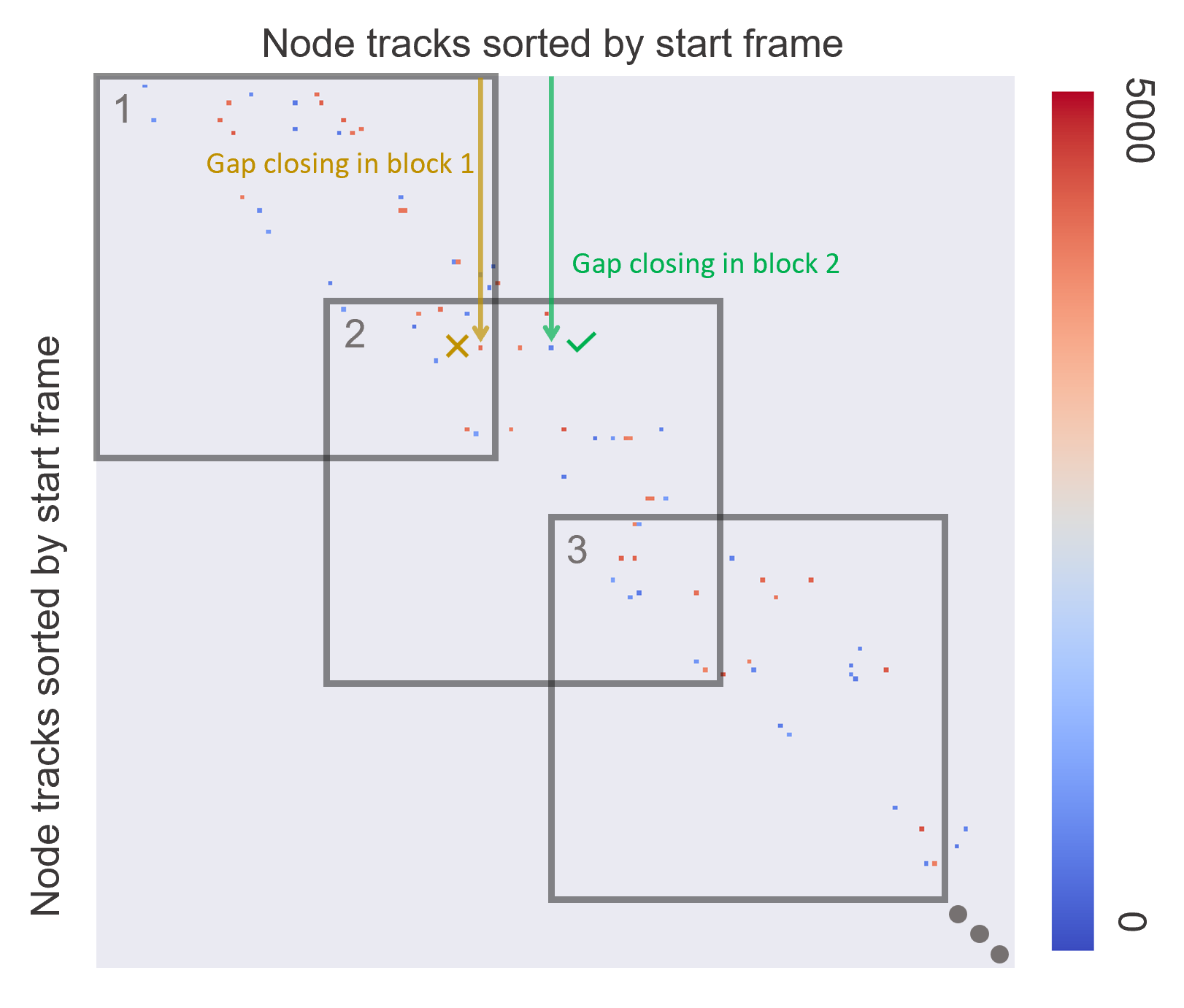

Supplement: S4 Fig — An illustrative gap closing matrix is shown. Row and column indices are node track IDs ranked by the track start frame number. The cost terms are calculated as the product of the distance and topology costs for the end node of the row track, and the start node of the column track. Thus, no assignments are allowed for the lower triangle. Because the number of tracks can be very large, overlapping blocks of the cost matrix are used for track assignments to reduce memory usage and speed up computation. Two track assignments for a track in the overlapped region between two blocks are shown. Because the track is on the edge of the block, the assignment from block 1 has high cost and is sub-optimal (yellow). However, because the block 2 includes more potential tracks, the assignment from block 2 gives the optimal track assignment. (TIFF) [file pcbi.1011060.s004.tiff]

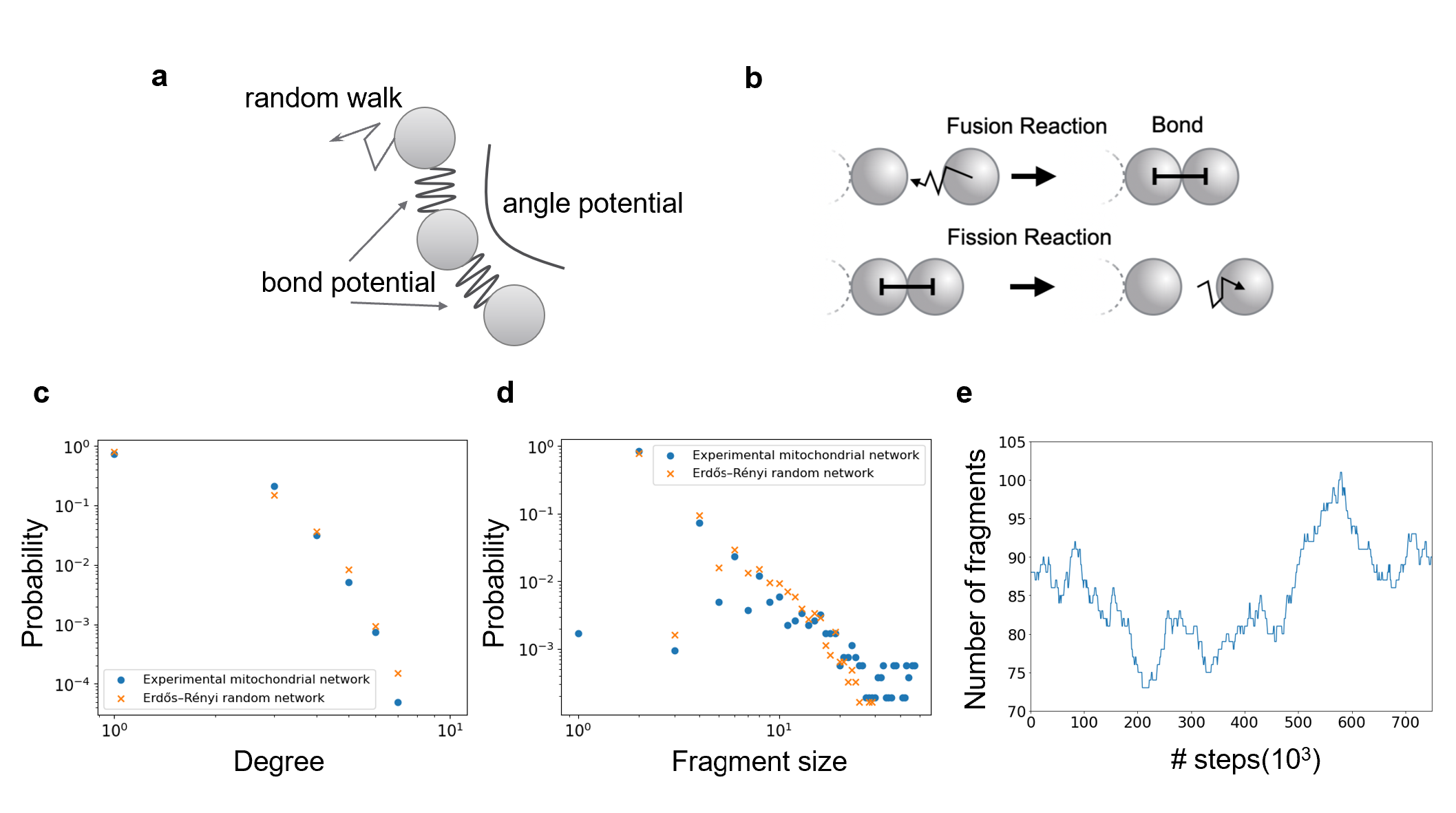

Supplement: S5 Fig — a, Mitochondrial network is modeled by diffusive skeleton particles constrained by angle and bond potentials. b, Fusion and fission events are modeled by random topology changing reactions. c-d, The degree distribution c) and the fragment size distribution d) for the experimental network segmented by MitoGraph (blue dot), and the random network generated for simulation (yellow cross). e, The total number of fragments in the simulation is monitored as a function of simulation timestep to ensure the balance between fusion and fission. (TIFF) [file pcbi.1011060.s005.tiff]

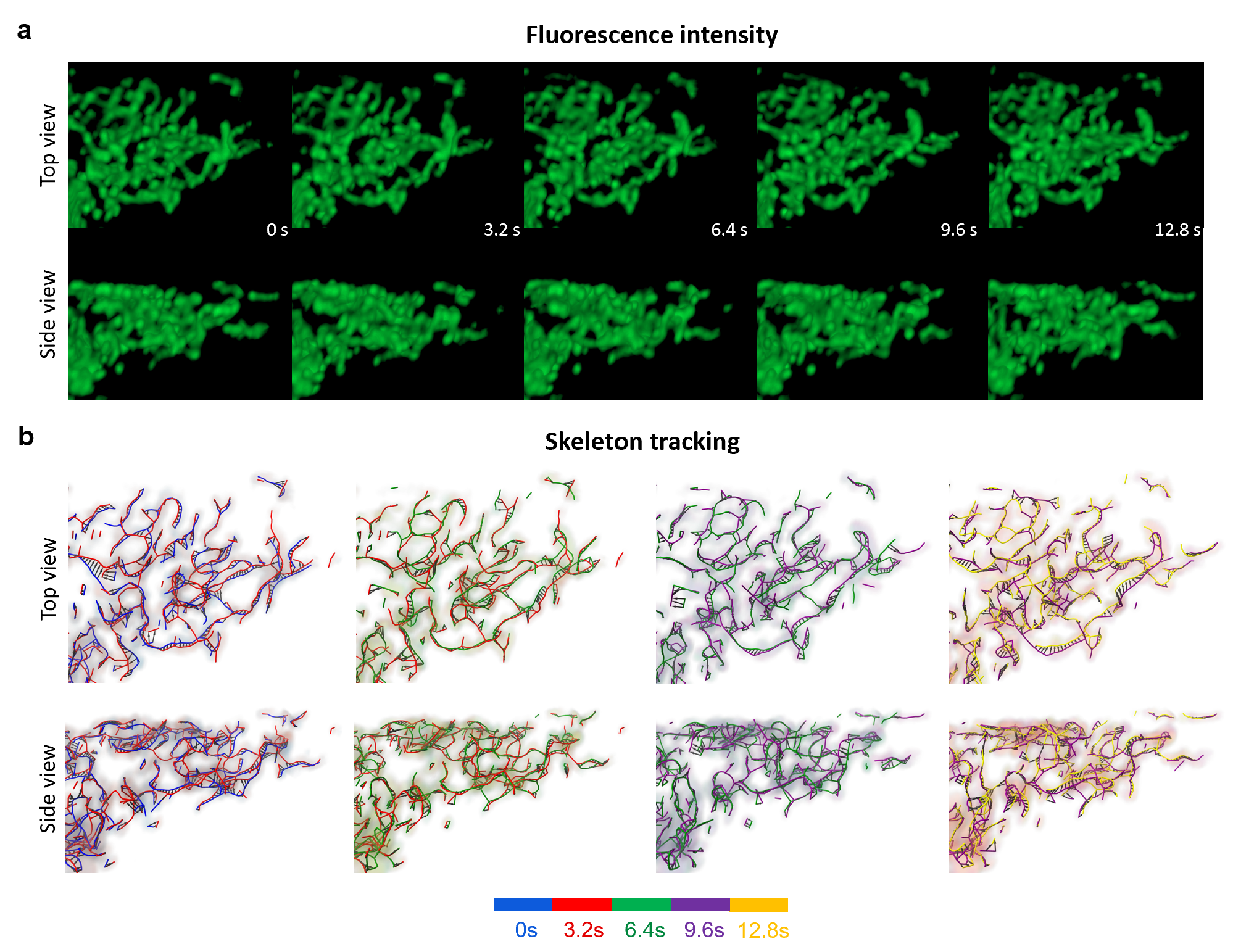

Supplement: S6 Fig — a, Fluorescence intensity over five frames. b, Network tracking for the region in a). (TIFF) [file pcbi.1011060.s006.tiff]

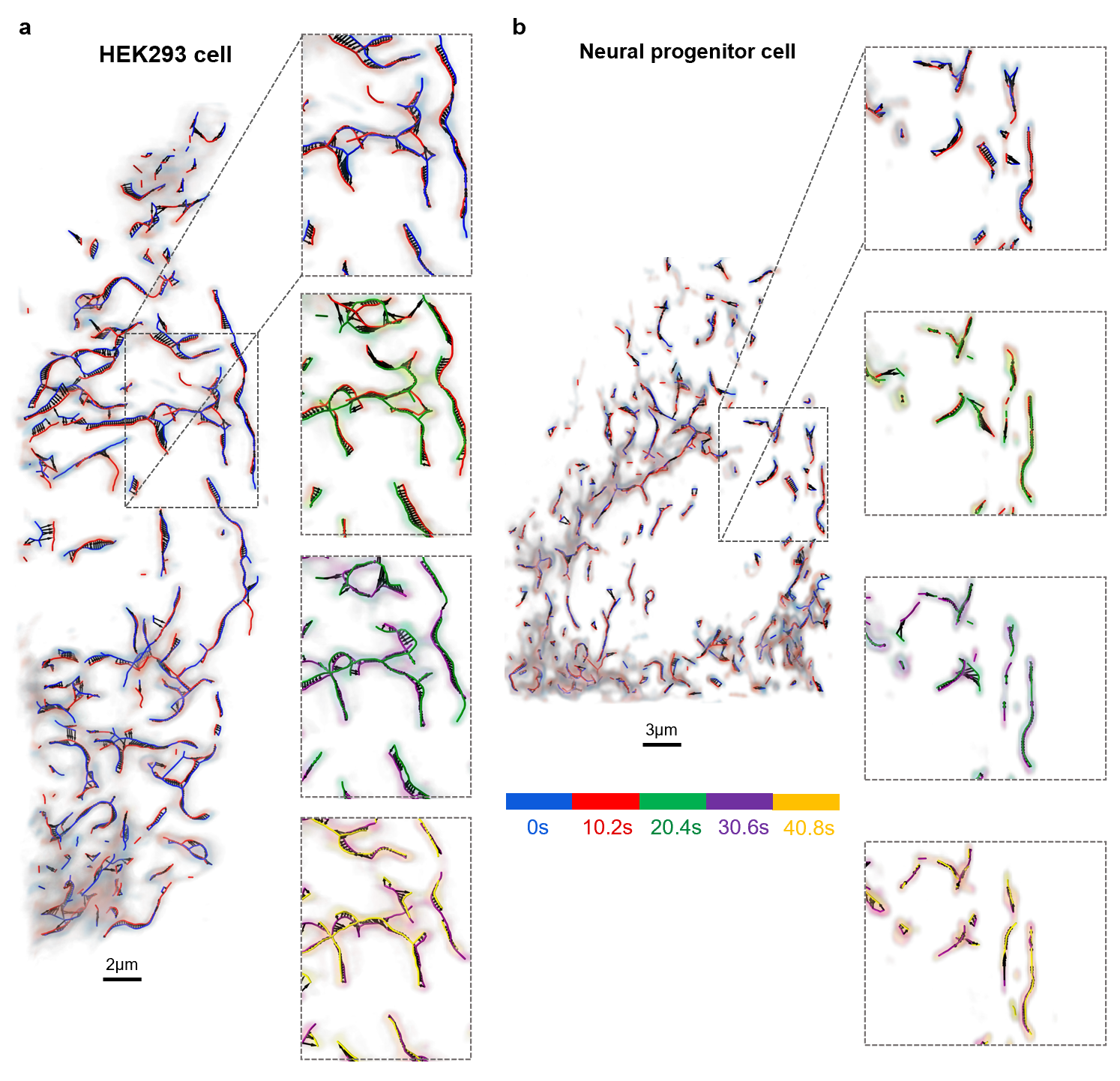

Supplement: S7 Fig — a-b, Fluorescence signal, segmented network skeleton, and tracking arrows are overlaid for two cell types other than hiPSC (HEK293 cell which is an immortalized cell line and NPC which is differentiated from hiPSC). One region in each cell type is zoomed in and tracked for 4 frames. (TIFF) [file pcbi.1011060.s007.tiff]

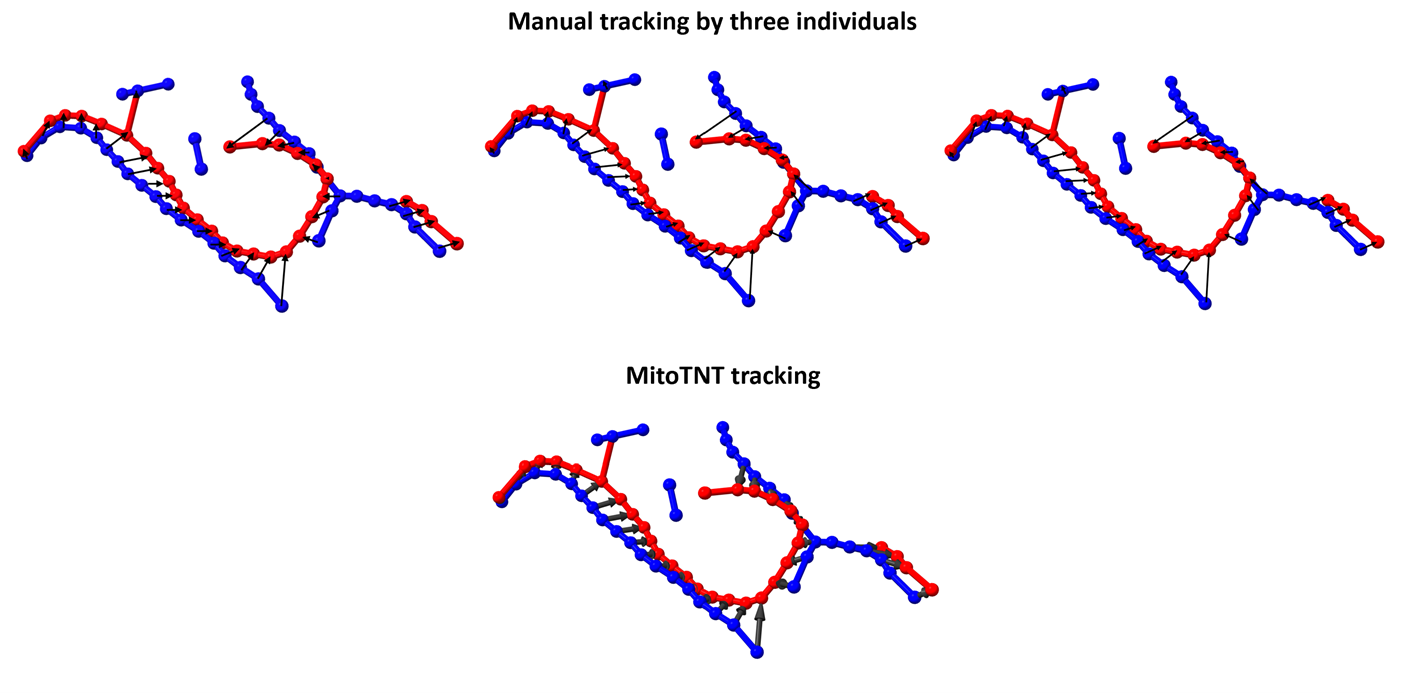

Supplement: S8 Fig — Manual tracking has been done independently by three individuals. One example tracking scenario is shown for manual tracking (top) and MitoTNT tracking (bottom). (TIF) [file pcbi.1011060.s008.tif]

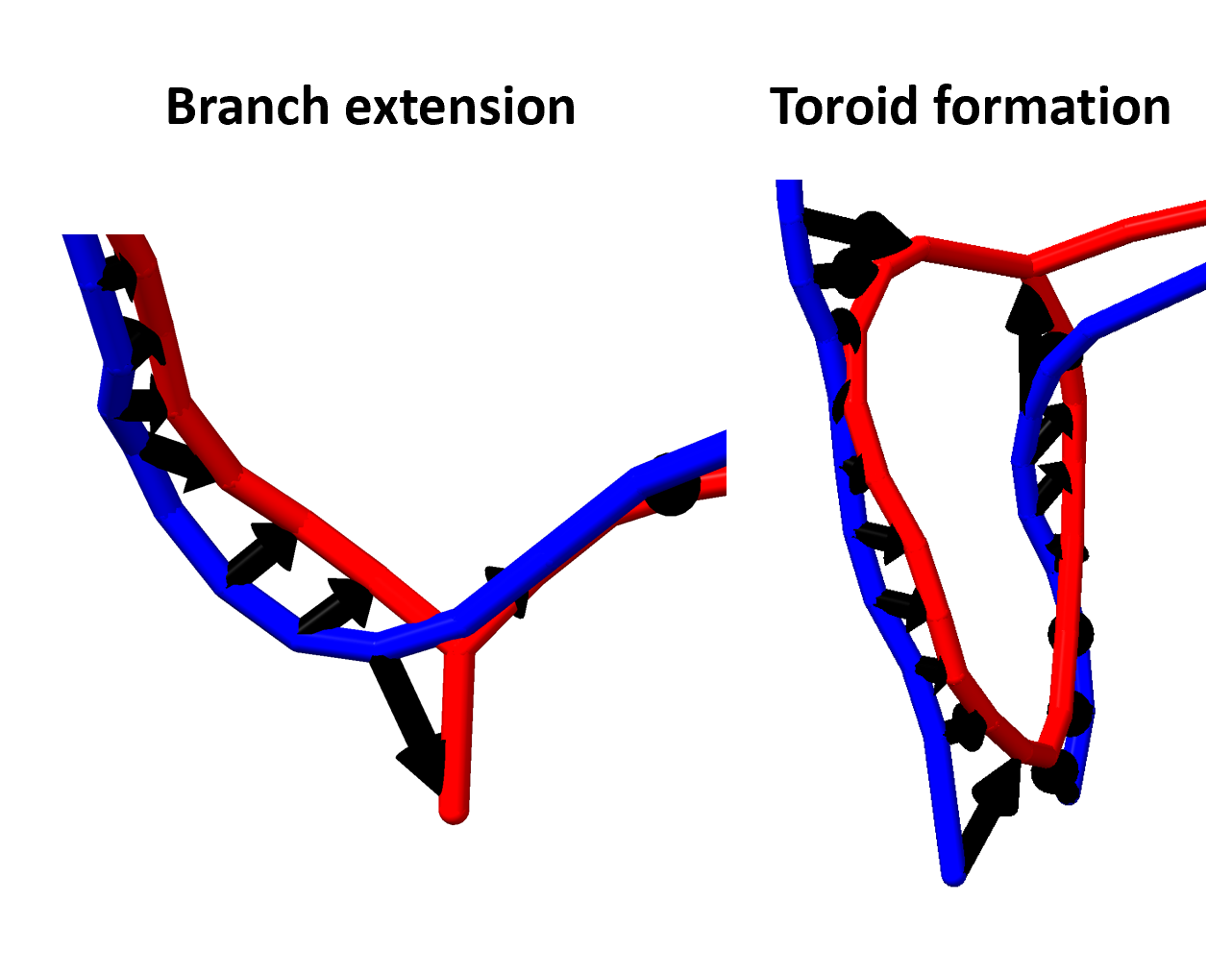

Supplement: S9 Fig — Segmented network skeleton for two consecutive frames and tracking arrows are plotted for a branch extension event (left) and for a toroid formation event (right). (TIFF) [file pcbi.1011060.s009.tiff]

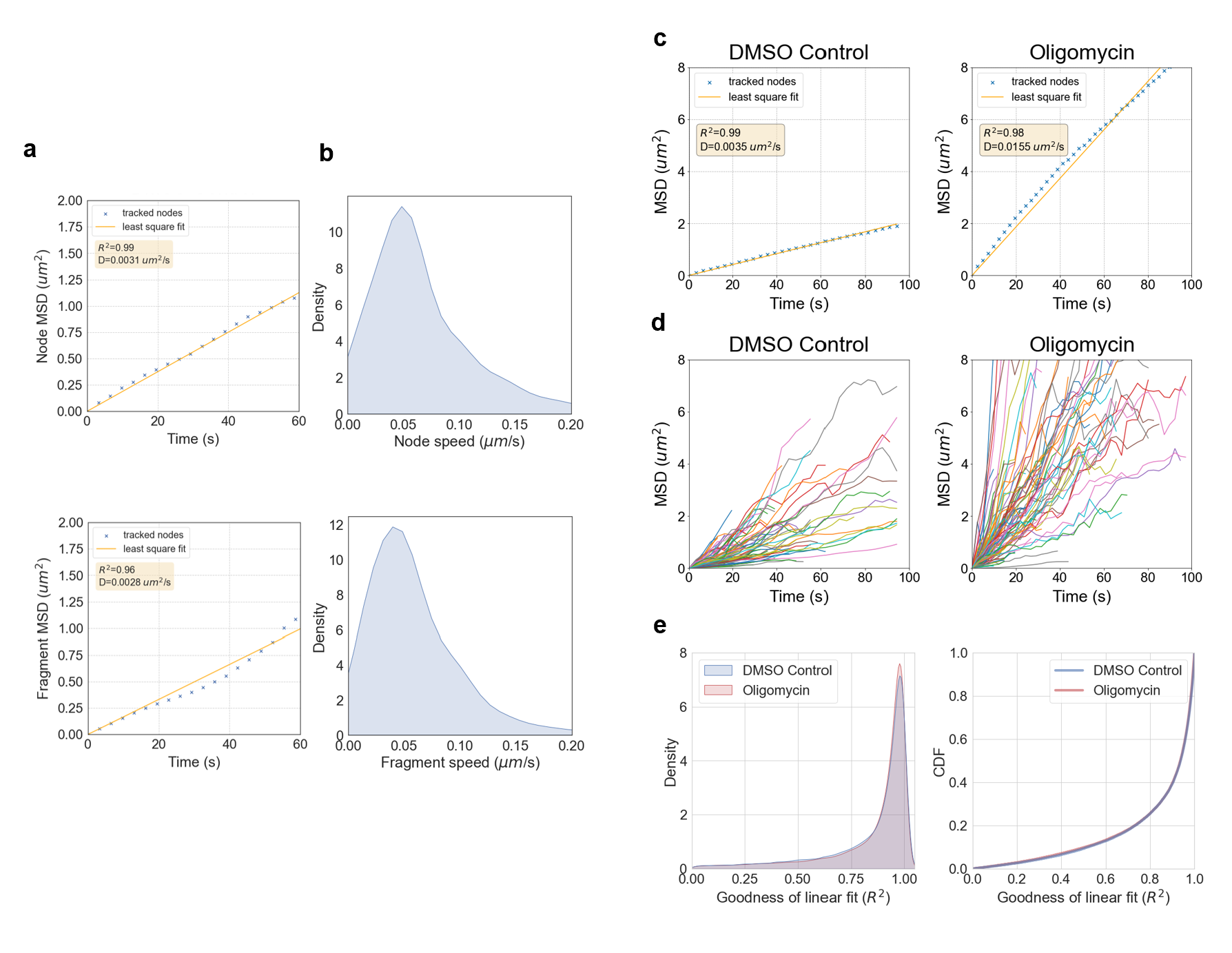

Supplement: S10 Fig — a, Mean square displacement (MSD) is computed with respect to time delays for skeleton nodes (top) and fragments (bottom). b, Diffusivity values in a) are plotted as a distribution for skeleton nodes (top) and fragments (bottom). c, Node-averaged and time-averaged mean square displacements (blue) are computed with respect to the time delays for two conditions, control and oligomycin. The linear fit line (orange) is shown together with the coefficient of determination (R2) and diffusion coefficient (D). d, Node-averaged MSD for individual tracks in control and oligomycin. e, The goodness of linear fit as measured by R2 is plotted as density distribution, and cumulative distribution function (CDF). R2 close to 1 indicates the data points follow a linear pattern. (TIFF) [file pcbi.1011060.s010.tiff]

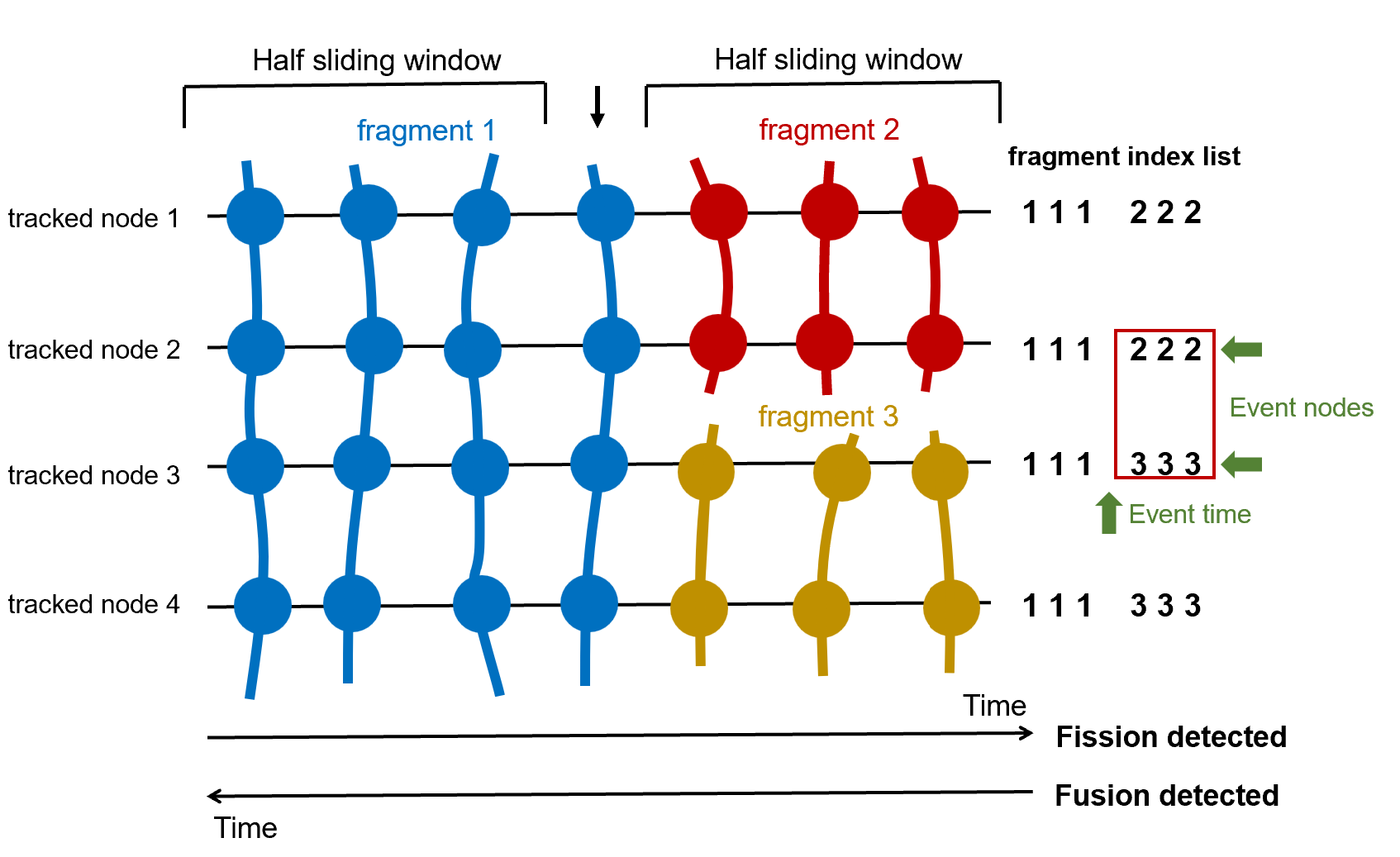

Supplement: S11 Fig — Four tracked nodes are positioned vertically, and seven timepoints are shown horizontally. The center of the sliding window is highlighted with the arrow on top. Three fragments are labeled and colored differently. The fragment indices for each node over time are stored. For each half-window, the index values between every two connected nodes are compared frame by frame. An event is declared if fragment indices in one sliding windows are strictly different in time, while those in the other sliding window are strictly identical in time. This requirement is imposed in order to avoid misidentifying transient segmentation noise as remodeling events. (TIFF) [file pcbi.1011060.s011.tiff]

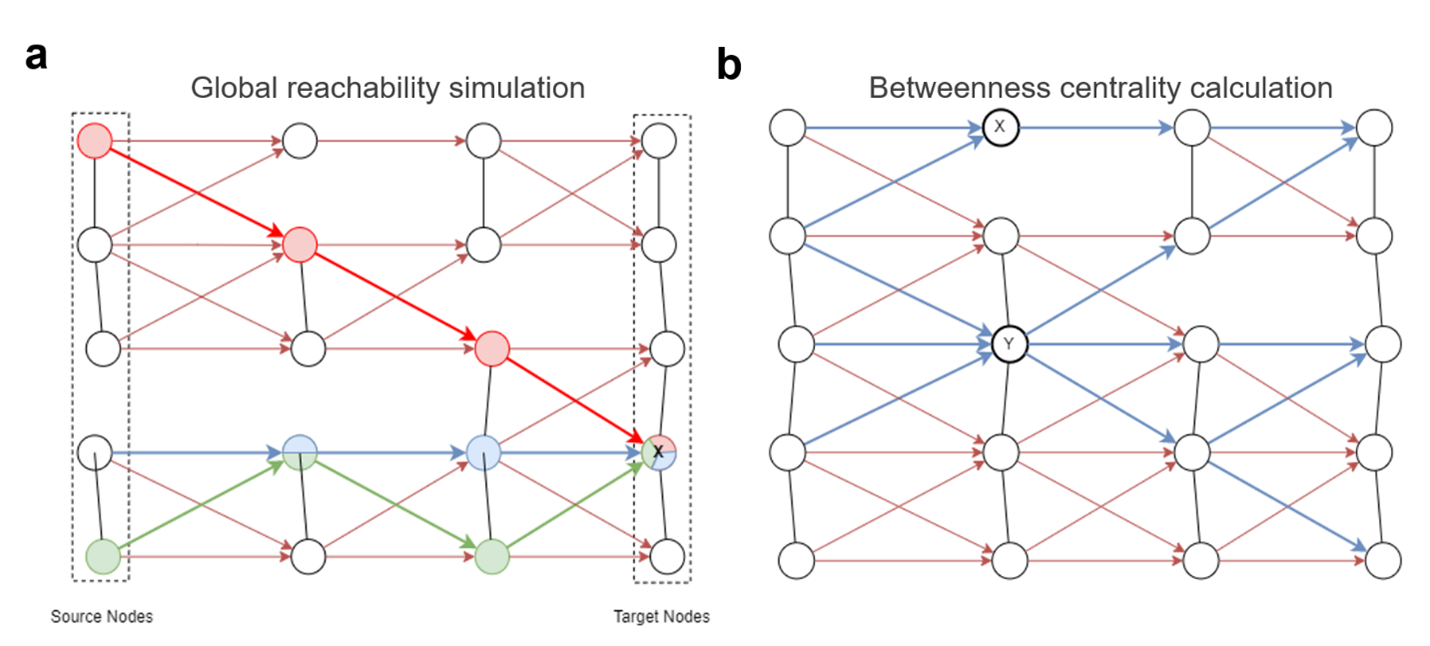

Supplement: S12 Fig — a, Illustration of the global reachability simulation. From each node, material can diffuse into the neighboring nodes or stay in the source node. All possible diffusion pathways over time are marked with an arrow. Time is depicted from left to right. Red, blue, and green arrows indicate representative simulation scenarios. After four timesteps, the target node labeled with X has accumulated three tokens (through the colored transport arrows). b, Illustration of the calculation for temporal betweenness centrality used for determining the central nodes. Each arrow represents the diffusion of material from one node at time t to the next node at time t+1. The temporal betweenness centrality of a node measures how many temporal shortest paths pass through that node. Node Y has a higher number of temporal shortest paths going through it than node X. Thus, node Y has a higher temporal betweenness centrality than node X. (TIF) [file pcbi.1011060.s012.tif]
